# DNMT3A and TET1 cooperate to regulate promoter epigenetic landscapes in mouse embryonic stem cells

**DOI:** 10.1186/s13059-018-1464-7

**Published:** 2018-07-12

**Authors:** Tianpeng Gu, Xueqiu Lin, Sean M. Cullen, Min Luo, Mira Jeong, Marcos Estecio, Jianjun Shen, Swanand Hardikar, Deqiang Sun, Jianzhong Su, Danielle Rux, Anna Guzman, Minjung Lee, Lei Stanley Qi, Jia-Jia Chen, Michael Kyba, Yun Huang, Taiping Chen, Wei Li, Margaret A. Goodell

**Affiliations:** 1 Stem Cells and Regenerative Medicine Center, Baylor College of Medicine, Houston, TX 77030 USA; 2 Program in Developmental Biology, Baylor College of Medicine, Houston, TX 77030 USA; 3 Medical Scientist Training Program, Baylor College of Medicine, Houston, TX 77030 USA; 4 Division of Biostatistics, Dan L. Duncan Cancer Center, Baylor College of Medicine, Houston, TX 77030 USA; 5 Department of Molecular and Cellular Biology, Baylor College of Medicine, Houston, TX 77030 USA; 6 Department of Bioinformatics, School of Life Sciences and Technology, Tongji University, Shanghai, China; 7 Department of Epigenetics and Molecular Carcinogenesis, The University of Texas MD Anderson Cancer Center, Smithville, TX 78957 USA; 8 Center for Epigenetics and Disease Prevention, Institute of Biosciences and Technology, Texas A&M University, Houston, TX 77030 USA; 9 Lillehei Heart Institute and Department of Pediatrics, University of Minnesota, Minneapolis, MN 55455 USA; 10 Department of Bioengineering, Stanford University, Stanford, California, USA; 11 Institute of Biochemistry and Cell Biology, Chinese Academy of Sciences, Shanghai, 200031 China

**Keywords:** DNMT3A, TET1, DNA methylation, H3K27me3, PRC2, Embryonic stem cells

## Abstract

**Background:**

DNA methylation is a heritable epigenetic mark, enabling stable but reversible gene repression. In mammalian cells, DNA methyltransferases (DNMTs) are responsible for modifying cytosine to 5-methylcytosine (5mC), which can be further oxidized by the TET dioxygenases to ultimately cause DNA demethylation. However, the genome-wide cooperation and functions of these two families of proteins, especially at large under-methylated regions, called canyons, remain largely unknown.

**Results:**

Here we demonstrate that DNMT3A and TET1 function in a complementary and competitive manner in mouse embryonic stem cells to mediate proper epigenetic landscapes and gene expression. The longer isoform of DNMT3A, DNMT3A1, exhibits significant enrichment at distal promoters and canyon edges, but is excluded from proximal promoters and canyons where TET1 shows prominent binding. Deletion of *Tet1* increases DNMT3A1 binding capacity at and around genes with wild-type TET1 binding. However, deletion of *Dnmt3a* has a minor effect on TET1 binding on chromatin, indicating that TET1 may limit DNA methylation partially by protecting its targets from DNMT3A and establishing boundaries for DNA methylation. Local CpG density may determine their complementary binding patterns and therefore that the methylation landscape is encoded in the DNA sequence. Furthermore, DNMT3A and TET1 impact histone modifications which in turn regulate gene expression. In particular, they regulate Polycomb Repressive Complex 2 (PRC2)-mediated H3K27me3 enrichment to constrain gene expression from bivalent promoters.

**Conclusions:**

We conclude that DNMT3A and TET1 regulate the epigenome and gene expression at specific targets via their functional interplay.

**Electronic supplementary material:**

The online version of this article (10.1186/s13059-018-1464-7) contains supplementary material, which is available to authorized users.

## Background

DNA methylation at the 5-position of cytosine (5mC) on CpG dinucleotides is a heritable epigenetic marker in mammals that is critical for development, X-chromosome inactivation, silencing of transposons and repeat elements; aberrant DNA methylation is often implicated in carcinogenesis [1, 2]. DNA methylation is generated by de novo methyltransferases 3A and 3B (DNMT3A and DNMT3B) and maintained by DNMT1. Mouse mutants lacking one or more DNMTs exhibit aberrant development [3, 4]. *Dnmt* triple knockout (TKO) embryonic stem cells (ESCs) progressively lose differentiation potential [5]. While DNA methylation is generally uniformly high throughout the genome (60–80% of CpGs), it is largely excluded from some regions, notably promoters, CpG islands (CGIs) and large under-methylated regions termed canyons (or valleys) [6, 7]. In addition, the pattern is broadly stable across the genome, except at certain regions such as enhancers [8] and canyon edges [6, 9]. The specific contributions of DNMTs to these dynamics and the mechanisms that exclude DNA methylation from certain regions are not well understood.

Ten-eleven translocation (TET) proteins have been identified as dioxygenases that convert 5mC to 5-hydroxymethycytosine (5hmC), 5-formylcytosine (5fC), and 5-carboxylcytosine (5caC) [10–12]. 5mC oxidation coupled with TDG-mediated base excision of 5fC or 5caC constitutes an active demethylation pathway [10]. TET proteins play important roles in ESC self-renewal and transcriptional regulation [13–15]. Both TET1 and TET2 are dispensable for embryonic development while TET3 is essential for oocyte reprogramming [16–18]. The roles of TET enzymes in the establishment and maintenance of the global DNA methylation pattern remain an area of intense research.

The genome-wide DNA methylation landscape changes dynamically during mammalian development [19]. Global waves of DNA demethylation mediated by TETs and re-methylation by DNMTs take place during early embryogenesis and gametogenesis. However, whether and how they function together to regulate DNA methylation, especially at specific genomic regions such as CGIs or canyons, has not yet been deeply investigated.

In the present work, we have taken advantage of mouse embryonic stem cells, where both DNMT3A/3B and TET1 are highly expressed, to elucidate the binding behaviors of DNMT3A and TET1 around transcriptional start sites (TSS) or canyons. We demonstrated that DNMT3A and TET1 impact gene expression via alterations in the histone landscapes surrounding these regions. In particular, they regulate gene expression at poised bivalent genes through affecting Polycomb Repressive Complex 2 (PRC2)-mediated H3K27me3 enrichment.

## Results

### Global DNA methylation in mouse ESCs is predominantly regulated by DNMT3A

**Fig. 1:**
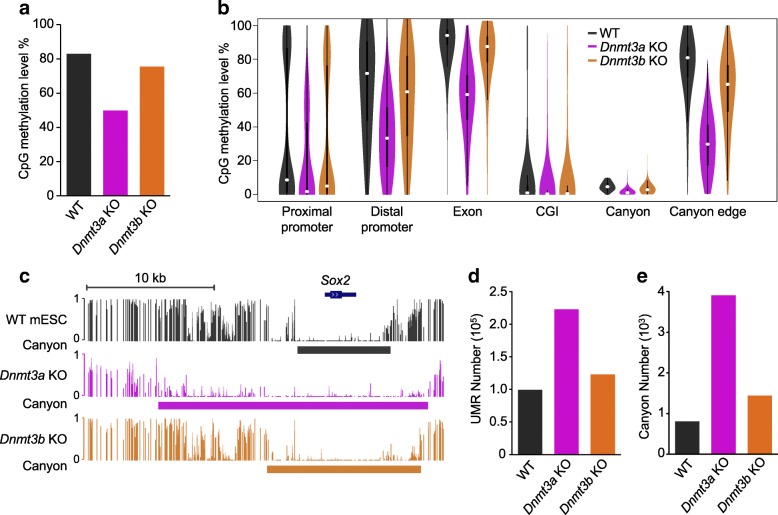
Global DNA methylation in mouse ESCs is predominantly regulated by DNMT3A. **a** Average genome-wide methylated CpG dinucleotides percentages in WT, *Dnmt3a* KO, and *Dnmt3b* KO ESCs. **b** *Violin plots* of CpG methylation levels in WT, *Dnmt3a* KO, and *Dnmt3b* KO ESCs determined by whole genome bisulfite sequencing (WGBS) at the indicated genomic features. **c** UCSC genome browser track depicting DNA methylation at the *Sox2* locus in WT, *Dnmt3a* KO, and *Dnmt3b* KO ESCs. Methylation ratios from 0 to 1 for individual CpG sites are shown. The identified DNA methylation canyon in each line is indicated by a *solid colored bar*. **d**, **e** The number of UMRs (**d**) and canyons (**e**) in the indicated lines of ESCs

To examine the distinct contributions of DNMT3A and DNMT3B to DNA methylation in mouse ESCs, we examined the patterns of DNA methylation after loss of *Dnmt3a* or *Dnmt3b*. DNA methylation landscapes at single-base resolution were generated by whole genome bisulfite sequencing (WGBS) between wild type (WT), *Dnmt3a* KO, and *Dnmt3b* KO J1 ESCs [3] with similar passage numbers. Over one billion sequencing reads were generated for each cell type, resulting in an average coverage of around 30-fold in each dataset. Although both methyltransferases are highly expressed and are known to contribute to maintenance of methylation genome-wide and at repetitive elements [3], loss of *Dnmt3a* had a much more dramatic impact than loss of *Dnmt3b* on DNA methylation genome-wide (Fig. 1a and Additional file 1: Figure S1a), with a significant decrease of CpG methylation level on distal promoters and at canyon [6] edges (Fig. 1b), as exemplified by the *Sox2* locus (Fig. 1c). The *Dnmt3a* KO ESCs also showed more significant reduction of non-CpG methylation (Additional file 1: Figure S1b). Compared to WT cells, the majority of differentially methylated regions (DMRs) in either KO cell line were hypomethylated (Additional file 1: Figure S1c), while a very small number of CpGs acquired methylation (Additional file 1: Figure S1d), likely due to aberrant activity of remaining methyltransferases or DNMT1. Consistent with the general hypomethylation, the number of short under-methylated regions (UMRs; 1 kb ≤ length < 3.5 kb, methylation ≤ 10%) approximately doubled in *Dnmt3a* KO cells (*n* = 22,280) compared to both WT (*n* = 9920) and *Dnmt3b* KO (*n* = 12,303) cells (Fig. 1d). In addition, the number of canyons (UMRs ≥ 3.5 kb) quadrupled in *Dnmt3a* KO cells (*n* = 3907, compared to 807 in WT, Fig. 1e). Together, these data demonstrate a predominant role of DNMT3A over DNMT3B in maintaining the global DNA methylation pattern in mouse ESCs.

### DNMT3A1 is enriched at distal promoter and canyon edges

**Fig. 2:**
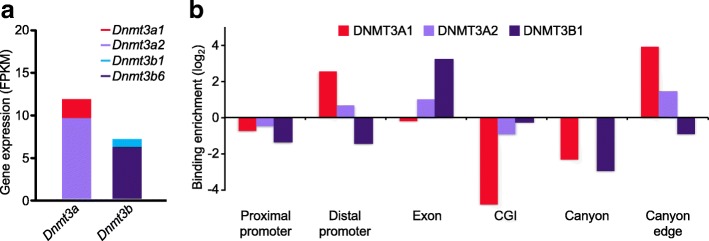
DNMT3A1 is enriched at distal promoter and canyon edges. **a** Expression levels (FPKM) determined by RNA-seq of major active isoforms of *Dnmt3a*: *Dnmt3a1* (NM_001271753, NM_007872), *Dnmt3a2* (NM_153743), and *Dnmt3b*: *Dnmt3b1* (NM_001003961), *Dnmt3b6* (NM_001003963) in WT J1 ESCs. **b** Relative binding enrichment between DNMTs (DNMT3A1, DNMT3A, and DNMT3B1) at different genomic features, compared to their average genomic distribution. The canyon edge corresponds to 2-kb flanking regions around canyons. DNMT3A2 and DNMT3B1 data are from [21]

We next sought to understand the relationship between the DNA methylation pattern and DNMT binding. At least two *Dnmt3a* and six *Dnmt3b* isoforms have been identified [20], among them *Dnmt3a2* and *Dnmt3b1* are catalytically active and strongly expressed in mouse ESCs. Previous work demonstrated DNMT3B1 preferentially binds within gene bodies of actively transcribed genes; both DNMT3A2 and DNMT3B1 are excluded from active promoters and enhancers [21]. The two isoforms of *Dnmt3a* originate from different promoters and differ by 219 N-terminal amino acids; both retain the catalytically active C-terminus [22]. Although DNMT3A2 predominates in ESCs, the longer isoform DNMT3A1 is still expressed (Fig. 2a). Thus, we explored the occupancy of DNMT3A1 on chromatin, trying to compare and reveal any binding difference between these two isoforms.

To examine the binding activity of DNMT3A1, we developed a Bio-*Dnmt3a1* ES cell line in a WT background that allows for doxycycline-inducible in vivo biotinylation of DNMT3A1. The generated cell line expresses an N-terminal Avi-tagged DNMT3A1 under control of a TetO promoter [23]. An IRES-biotin ligase (BirA) sequence included in the vector allows for biotin tagging at the N-terminus of DNMT3A1. After confirming induction of the biotinylated-DNMT3A1 protein (Additional file 1: Figure S2a), genome-wide binding maps of DNMT3A1 were generated by biotin-based chromatin immunoprecipitation (ChIP) followed by high-throughput sequencing. DNMT3A1, and to a lesser extent DNMT3A2, is preferentially enriched in distal promoter regions (500–3000 bp upstream of TSSs), differing from the specific genic enrichment within exon/gene bodies, as observed for DNMT3B1 (Fig. 2b and Additional file 1: Figure S2b). Strikingly, DNMT3A1 and DNMT3A2 are most enriched at canyons edges (2 kb flanking both sides of a canyon, Fig. 2b and Additional file 1: Figure S2c). DNMT3A2 also binds in gene bodies. All DNMTs, with DNMT3A1 to the largest extent, are excluded from regions known to have low methylation, such as proximal promoters (TSS ± 500 bp), CGIs, and canyons themselves (Fig. 2b). It has been reported that both DNMT3A2 and DNMT3B1 prefer methylated CpG-rich regions, with a drop off at CGIs [21]. DNMT3A1 exhibits a trend very similar with DNMT3A2 and DNMT3B1. However, there is more DNMT3A2 and DNMT3B1 binding at regions with higher CpG density where DNMT3A1 appears to drop off sharply (Additional file 1: Figure S2d). Our data suggested that DNMT3A1 has unique properties in genome binding and may have special functions in regulating DNA methylation and gene expression. These clear distinctions led us to focus on the potential roles of DNMT3A1 at distal promoters and canyons.

### DNMT3A1 and TET1 have complementary binding patterns

We had previously shown in mouse hematopoietic stem cells (HSCs) that 5hmC accumulates at the edges of canyons; we proposed that the TET family of proteins (TET1/2/3), which catalyze conversion of 5mC to 5hmC, may be localized to these sites [6]. To test this hypothesis, we examined the binding relationship between DNMT3A1 and TET1, the TET family member most highly expressed in mouse ESCs. To determine the binding pattern of TET1 genome-wide, CRISPR/Cas9-facilitated homology directed repair (HDR) was utilized to develop an ESC line that contains a C-terminal 3 × FLAG tag on both alleles (*Tet1*-FLAG ESCs, Additional file 1: Figure S3a). Similar 5mC and 5hmC levels were observed by dot blot with genomic DNA from WT and *Tet1*-FLAG ESCs, indicating proper enzymatic activity of TET1-FLAG protein (Additional file 1: Figure S3b–e). We also performed ChIP-seq with *Tet1*-FLAG ESCs using anti-FLAG M2 and an anti-TET1 antibody [24]. A strong correlation between the two datasets suggested that the C-terminal tag did not interfere with the genome localization of TET1 and suggested we could accurately measure TET1 protein distribution on chromatin (Additional file 1: Figure S4a).

**Fig. 3:**
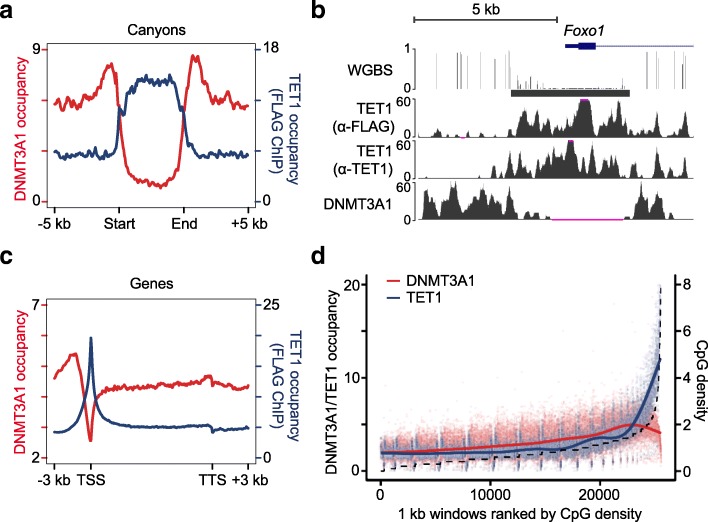
DNMT3A1 binding is complementary to TET1. **a** Average binding occupancy of DNMT3A1 and TET1 (anti-FLAG ChIP-seq) across all canyons, normalized to same length (Start-End), and 5-kb flanking regions. **b** UCSC genome browser track depicts DNA methylation, TET1 (anti-FLAG and anti-TET ChIP), and DNMT3A1 binding profiles around *Foxo1* gene promoter in *Tet1*-FLAG ESCs. **c** Average binding occupancy of DNMT3A1 and TET1 (anti-FLAG ChIP-seq) across all gene bodies and 3-kb flanking regions. **d** DNMT3A1 and TET1 binding enrichment in 1-kb genomic windows ranked by CpG density, which was displayed as a dashed line. The point where DNMT3A and TET1 lines cross on right *y-axis* represents roughly 1.5 CpG per 100 bp

The binding pattern of TET1 was highly complementary to DNMT3A1 at specific genomic regions, particularly canyons and TSSs. TET1 binds throughout canyons, while DNMT3A1, excluded from binding within the canyon itself, exhibits peak binding at the canyon edges (Fig. 3a and Additional file 1: Figure S4b). As exemplified at the *Foxo1* locus, TET1 is seen binding in the canyon from edge-to-edge, whereas DNMT3A1 accumulates at the edges and outside the canyon (Fig. 3b). In addition, at TSSs, where TET1 is known to accumulate [14, 15, 24], this opposing binding pattern was similarly observed (Fig. 3c and Additional file 1: Figure S4c). Broadly, these observations are consistent with a reciprocal relationship of these proteins in their ability to interact with DNA containing different levels of CpG density. Specifically, as CpG density increases > 1.5 CpG per 100 bp, TET1 binding dramatically increases, while DNMT3A1 binding diverges in the opposite direction (Fig. 3d), suggesting that CpG density might influence the complementary genomic occupancy of DNMT3A1 and TET1. Indeed, previous studies reported that the genomic binding of TET1 is positively correlated with CpG density [15].

### DNMT3A and TET1 binding correlate with histone marks and overall gene expression

**Fig. 4:**
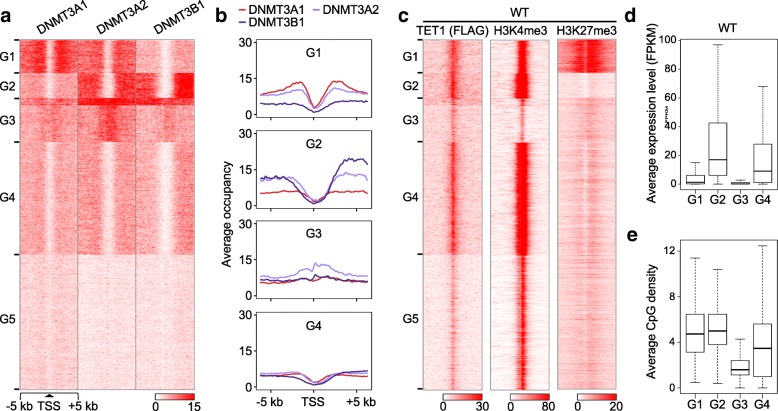
DNMT3A and TET1 binding correlate with histone marks and overall gene expression. **a** *Heatmaps* showing DNMT3A1, DNMT3A2, and DNMT3B1 binding around all TSSs in mouse genome which were clustered into five groups (G1–G5) based on their different binding properties in WT ESCs. **b** Quantification of (a): Average occupancy of DNMT3A1, DNMT3A2, and DNMT3B1 at genes from G1–G4. **c** *Heatmaps* showing TET1 and histone marks (H3K4me3 and H3K27me3) enrichment in WT ESCs around all TSSs, listed in the same order as Fig. 4a. **d** *Boxplots* of gene expression (FPKM) from G1–G4 in WT ESCs. **e** *Boxplot* of CpG density (normalized to 100 bp) around gene promoters (− 1 kb ~ + 0.5 kb) in G1–G4

To further understand the impact of DNMT3A and TET1 on the other protein’s binding capacity, we looked more closely at different TSS regions and their relationships with the DNMT3B and the DNMT3A isoforms in WT ESCs. First, we clustered all of the TSSs in the mouse genome into five groups based on the binding signals of DNMT3A1, 3A2, and 3B1 (Fig. 4a, b). We focused on Groups 1–3 (G1, G2, and G3) because they exhibited relatively higher occupancy of DNMTs and/or TET1 (Fig. 4a–c). DNMT3A1 specifically binds around the TSSs of G1 genes. In contrast, DNMT3B1 is preferentially enriched in the gene bodies of G2 genes. DNMT3A2 co-binds with DNMT3A1 at G1 genes and together with DNMT3B1 at G2 genes (Fig. 4a, b). Therefore, DNMT3A2 may compensate for the function of DNMT3B1 in *Dnmt3b* KO cells; however, DNMT3A function cannot be fully compensated by DNMT3B1 in *Dnmt3a* (both *Dnmt3a1* and *Dnmt3a2*) KO cells, resulting in significant loss of DNA methylation (Fig. 1). Surprisingly, we also found DNMT3A, especially DNMT3A2, binds within the TSSs of G3 genes (Fig. 4a). These results confirmed that DNMT3A and DNMT3B have distinct binding properties and further distinguished the isoform-specific binding patterns of DNMT3A1 and DNMT3A2.

In order to understand how DNMT3A and TET1 influence other epigenetic markers, we examined the histone landscape and gene expression levels in WT cells for each group (Fig. 4c, d). Importantly, we discovered that G1 genes possess high levels of both H3K4me3 and H3K27me3, so called bivalent genes [25], suggesting that DNMT3A1 and TET1 may bind and function together to regulate the expression of bivalent genes in ESCs. These data are also consistent with recent findings that DNMT3A1 shows significant enrichment around bivalent genes [26]. G2 genes exhibit high levels of H3K4me3 and TET1, low levels of H3K27me3 at TSSs and high DNMT3B1 binding in flanking regions; accordingly, they are the most highly expressed gene group in mouse ESCs (Fig. 4c, d). In contrast, G3 genes carry the lowest level of H3K4me3 and TET1, while at the same time their TSSs are bound by DNMT3A and are heavily methylated (Additional file 1: Figure S5), probably because of lower CpG density at these TSSs [27] (Fig. 4e). Correspondingly, they have the lowest overall expression (Fig. 4d). While the converse of the majority of TSSs, which typically exhibit high TET1 binding, the reciprocal binding pattern of DNMT3A and TET1 is still observed in G3. Thus, the binding pattern of DNMT3A and TET1 correlates very well with histone landscapes and gene expression in WT ESCs.

### TET1 protects from DNMT3A1 binding to limit DNA methylation

The notable exclusion of DNMT3A1 binding to TET1-occupied genomic regions strongly suggests a functional relationship between TET1 and DNMT3A binding to DNA. Previous studies have shown that depletion of TET1 via RNA interference (RNAi) can lead to a slight global increase in the level of 5mC, as well as localized increase in 5mC at TSSs and the genomic regions flanking their proximal promoters [15, 28]. To determine the degree to which loss of TET1 permits unabrogated access of DNMT3A1, we generated Bio-*Dnmt3a1* - *Tet1* KO ES cell lines by utilizing CRISPR/Cas9 and a single guide RNA (sgRNA) previously described [29]. After confirming lack of TET1 protein and induced expression of Bio-DNMT3A1 (Additional file 1: Figure S6a, b), global changes of both 5mC and 5hmC were determined in the established cell lines. Dot blot analysis verified increased global 5mC and decreased 5hmC (Additional file 1: Figure S6c, d) in *Tet1* KO ESCs.

**Fig. 5:**
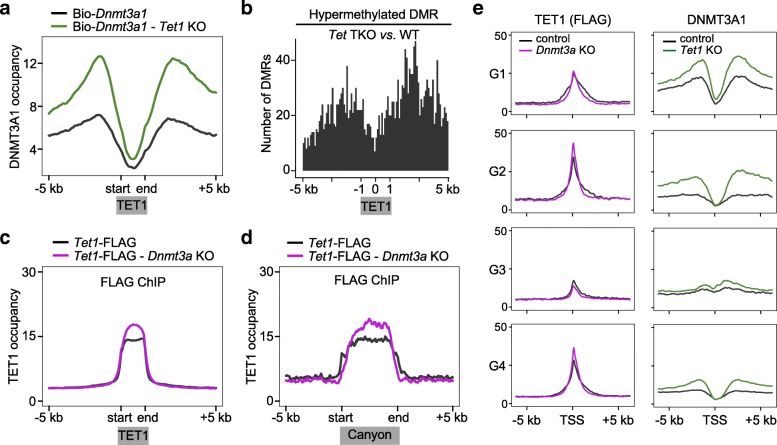
DNMT3A and TET1 impact each other’s binding on chromatin. **a** The average occupancy of DNMT3A1 in Bio-*Dnmt3a1* and Bio-*Dnmt3a1* - *Tet1* KO ESCs on TSS-associated TET1 peaks and the flanking 5-kb regions. **b** Position of hypermethylated DMRs around TSS-associated TET1 peaks, comparing *Tet1/2/3* TKO (*Tet* TKO) [30] and WT ESCs. The normalized TET1 peaks are positioned with 0 representing the peak centers, and positions ± 1 representing the TET1 peak edges, as indicated by the *gray bar*. **c**, **d** The average occupancy of TET1 (anti-FLAG ChIP-seq) in *Tet1*-FLAG and *Tet1*-FLAG - *Dnmt3a* KO ESCs across all TET1 peaks (**c**) and canyons (**d**). **e** Average occupancy of TET1 in *Tet1*-FLAG and *Tet1*-FLAG - *Dnmt3a* KO ESCs and DNMT3A1 in WT (Bio-*Dnmt3a1*) and *Tet1* KO ESCs at genes from each group

We next determined the distribution of DNMT3A1 in the absence of TET1 by biotin-based ChIP-seq. DNMT3A1 binding capacity increased dramatically around the TET1 binding peaks identified in WT cells (TSS associated), both in overall occupancy (peak height) and by invading into flanking regions (peak width, Fig. 5a). The increased and expanded binding by DNMT3A1 strikingly mirrors the sites of augmented DNA methylation observed in *Tet1/2/3* Triple KO (*Tet*-TKO) ESCs [30] (Fig. 5b). We further examined all TSS regions, comparing DNMT3A1 binding in WT and *Tet1* KO ESCs, ranking them by the magnitude of change in DNMT3A1 occupancy after *Tet1* KO. The regions with the greatest change were also those with highest TET1 binding in WT ESCs (Additional file 1: Figure S7a). DNMT3A1 binding is also anticorrelated with the extent of H3K4me3 levels (Fig. 4a, c). However, loss of TET1 has little effect on H3K4me3 level at TSSs (Additional file 1: Figure S7b, c), making it unlikely that H3K4me3 could impact DNMT3A binding in *Tet1* KO ESCs. Collectively, the presence of TET1 constrains DNMT3A1 binding in CpG-rich environments, such as proximal promoter regions, inhibiting DNA methylation.

### *Dnmt3a* ablation has a weak but promotor-specific impact on TET1 distribution

The finding that TET1 limits DNMT3A’s access to TSSs and canyons raised the possibility that DNMT3A may reciprocally constrain TET1. To test this possibility, we deleted *Dnmt3a* (both *Dnmt3a1* and *Dnmt3a2*) in *Tet1*-FLAG ESCs (Additional file 1: Figure S7d) using CRISPR/Cas9-facilitated knockout and then performed FLAG ChIP-seq in *Dnmt3a* KO; *Tet1*-FLAG ESCs. Similar to the behavior of DNMT3A1 following *Tet1* deletion, there was increased TET1 occupancy genome-wide within both TET1 peaks and canyons in *Dnmt3a* KO cells, compared with the parental *Tet1*-FLAG ESCs (Fig. 5c, d). However, the magnitude of these changes was modest compared to the experiments described above when *Tet1* was deleted. The slightly increased binding of TET1 was limited largely to peak height, with no significant increase in peak width, indicating there was little expansion of access to TET1 following *Dnmt3a* deletion. The same trend was observed by ChIP-seq with anti-TET1 antibody using the same cells (Additional file 1: Figure S7e, f).

Next, TET1 and DNMT3A1 binding in KO and control cells were displayed based on the same clustering used in Fig. 4 (Additional file 1: Figure S7g and Fig. 5e). Deletion of *Tet1* results in increased binding by DNMT3A1 in all groups, but at a higher level in G1 and G2 genes. On the other hand, deletion of *Dnmt3a* leads to a moderate increase in binding of TET1 at TSSs of genes from G2 and G4, but a more centralized binding at TSSs from G1 genes, where DNMT3A1 specifically binds (Fig. 4a). Although TET1 binding was affected differently within different groups, it generally increased after *Dnmt3a* deletion. Nevertheless, the 5hmC level was lower at genes from all groups, resulting from a significantly reduced level of 5mC (Additional file 1: Figure S5), the substrate for TET proteins to generate 5hmC [12].

It has been reported that TET-mediated DNA demethylation mainly occurs at promoters and enhancers [30]. We examined the binding pattern of TET1 at enhancers and surprisingly found that TET1 occupancy at enhancer regions decreased upon *Dnmt3a* deletion (Additional file 1: Figure S8a), which is distinct from the change at promoters. Furthermore, TET1 abundance is relatively low at enhancers with high levels of H3K27ac and H3K4me, correlating with lower CpG density at these stronger enhancers (Additional file 1: Figure S8b).

Together, these data demonstrate the clear reciprocal nature of DNMT3A1 and TET1 binding. While TET1 binding is altered after loss of DNMT3A, it does not extend outside of the canyon boundaries substantially. This suggests TET1 may be restrained within regions of the highest CpG density, thereby allowing it to act as an anchor, impeding access of DNMT3A to limit DNA methylation.

### DNMT3A and TET1 dynamically regulate gene expression

As discussed above, the patterns of histone marks and overall gene expression differ according to DNMT3A and TET1 binding in WT cells. To further elucidate the impact of these proteins on gene expression, we regenerated *Dnmt3a* KO, *Tet1* KO, and *Dnmt3a-Tet1* Double KO (DKO) cells from WT J1 ESCs and assessed changes of expression by RNA-sequencing (RNA-seq).

**Fig. 6:**
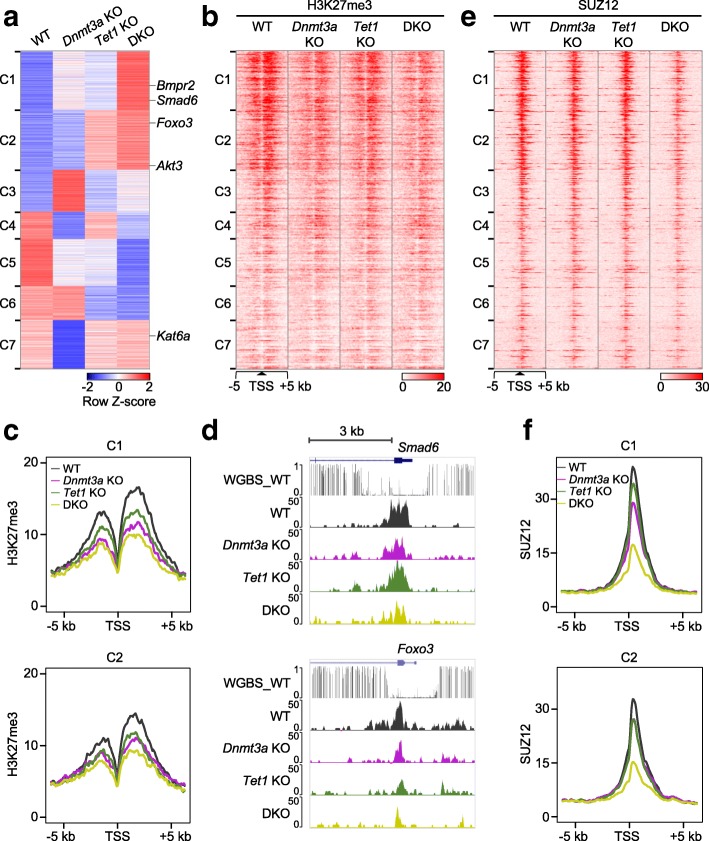
DNMT3A and TET1 regulate bivalent gene expression via PRC2-mediated H3K27me3. **a** Hierarchical clustering analysis of differentially expressed genes in WT, *Dnmt3a* KO, *Tet1* KO, and *Dnmt3a*-*Tet1* DKO ESCs. Genes *Smad6* and *Bmpr2* belong to Hippo / TGF-beta signaling (over-represented in C1) while *Foxo3* and *Akt3* in PI3K-Akt signaling pathway (over-represented in C2). Their expression difference between cell lines was confirmed by RT-qPCR (shown in Additional file 1: Figure S9b). **b** *Heatmap* representations of H3K27me3 enrichment on the flanking 5 kb surrounding the specific TSSs in each cluster in WT, *Dnmt3a* KO, *Tet1* KO, and DKO ESCs. All TSSs are listed in the same order as Fig. 6a. **c** Average density profiles for H3K27me3 marks at genes from C1 (*top*) and C2 (*bottom*) clusters in indicated cells. **d** UCSC genome browser tracks depicting DNA methylation (WGBS) in WT ESCs and H3K27me3 in WT, *Dnmt3a* KO, *Tet1* KO, and DKO ESCs at *Smad6* (*top*) and *Foxo3* gene (*bottom*). **e** Heatmap representations of SUZ12 occupancy on the flanking 5 kb surrounding the TSSs in cluster C1–C7 in WT, *Dnmt3a* KO, *Tet1* KO and DKO ESCs. **f** Average density profiles for SUZ12 occupancy at genes from C1 (*top*) and C2 (*bottom*) clusters in indicated cells

We identified 3007 genes as differentially expressed between WT, *Dnmt3a* KO, *Tet1* KO, and DKO cells. Seven clusters of genes were classified (C1–C7) based on their expression changes compared to WT. Generally, these differentially expressed genes (DEGs) were either specific to *Dnmt3a* deletion (C3, C4, and C7) or *Tet1* deletion (C2 and C6), or changed commonly by both, mostly in the same directions (C1 and C5, Fig. 6a). It was surprising that so few genes were changed in opposing directions in response to *Dnmt3a* KO or *Tet1* KO, considering the opposing enzyme activities (methylation and demethylation) of the two proteins. Compared with WT, many genes (C4, C5, and C7, 39% of all DEGs) were downregulated in *Dnmt3a* KO cells, suggesting the correlation between DNA methylation and gene repression is low. Enrichment analysis (Gene Ontology) showed that genes from Hippo and TGF-beta signaling pathway were over-represented in C1, while PI3K-Akt signaling pathway genes were enriched in C2 (Additional file 1: Figure S9a). Interestingly, for genes from C1 and C5, the expression change caused by *Dnmt3a* KO and *Tet1* KO was additive, resulting in the greatest bigger change in DKO cells (Fig. 6a). The expression changes of representatives from these pathways was further confirmed by quantitative reverse transcription polymerase chain reaction (RT-qPCR) (Additional file 1: Figure S9b). These data suggest that gene expression in mouse ESCs is dynamically regulated by DNMT3A or/and TET1, and support the concept that DNMT3A and TET proteins can cooperate at specific loci to regulate gene expression [31].

### DNMT3A and TET1 constrain expression of bivalent genes, likely via PRC2-mediated H3K27me3

Next we analyzed the histone marks (H3K4me3 and H3K27me3) in the various KO cell lines at differentially expressed genes (Fig. 6b and Additional file 1: Figure S10a) and compared them with those of WT cells (Additional file 1: Figure S10b). Remarkably, we found for each cluster of genes, that gene expression changes could be explained by histone modification changes. For example, C3 genes were upregulated in *Dnmt3a* KO cells in concert with increased H3K4me3. Concomitantly, C7 genes were downregulated along with decreased H3K4me3. C5 genes were downregulated in all KO cells, accompanied by increased H3K27me3 and decreased H3K4me3 (Additional file 1: Figure S10b). We focused on C1 and C2 because many genes in the two clusters carry both H3K4me3 and H3K27me3 (Additional file 1: Figure S10a and Fig. 6b). As discussed above, genes in C1 and C2 are DNMT3A- (especially DNMT3A1) and TET1-specific/enriched bivalent genes (Fig. 4 and Additional file 1: Figure S10c). Loss of DNMT3A and/or TET1 at C1 genes led to increased gene expression. This upregulation in gene expression was associated with diminished H3K27me3 levels, to a greater extent in *Dnmt3a* KO and DKO cells (Fig. 6a–c and Additional file 1: Figure S10b), as exemplified by the *Smad6* gene (Fig. 6d, Additional file 1: Figure S9b and S10d). Despite similar overall changes to histone marks seen at C1 genes, particularly with respect to H3K27me3, expression levels of C2 genes were largely unaffected by *Dnmt3a* deletion (Fig. 6a–c); this is exemplified by the *Foxo3* gene (Fig. 6d, Additional file 1: Figure S9b and S10d).

Because the interplay between DNMT3A and TET1 appeared focused on genes with bivalent histone marks, we examined the impact of DNMT3A and/or TET1 deficiency on the PRC2, which is responsible for establishing the H3K27me3 marks. We checked the expression of *Ezh2* and *Suz12*, two core components of PRC2 complex, and binding activity of SUZ12, comparing *Dnmt3a* KO, *Tet1* KO, and DKO with control cells. No change in expression of these core components was detected in KO cells (Additional file 1: Figure S10e); however, enrichment of SUZ12 at both C1 and C2 genes was reduced, to the greatest extent in DKO cells (Fig. 6e, f and Additional file 1: Figure S10f), which was coincident with the greatest degree of H3K27me3 reduction. These results suggest that DNMT3A and TET1 binding at and around specific TSSs functions to mediate binding, and thus overall activity, of the PRC2 complex, ultimately regulating gene expression from bivalent promoters.

To further verify our findings that DNMT3A and TET1 synergistically regulate H3K27me3 deposition at bivalent promoters, we performed a normalized histone ChIP-seq by adding spike-in chromatin and antibody in the reactions [32]. Similar results were obtained from the spike-in ChIP-seq: H3K27me3 decreased at DEG clusters C1 and C2 in KO cells, especially DKO cells, while H3K4me3 became lower at C7 genes in *Dnmt3a* KO cells (Additional file 1: Figure S11a, b). These results were confirmed by ChIP-qPCR normalized to a positive control in spike-in chromatin (Additional file 1: Figure S11c, d).

### DNMT3A and TET1 regulated H3K27me3 enrichment may be partially DNA methylation-independent

**Fig. 7:**
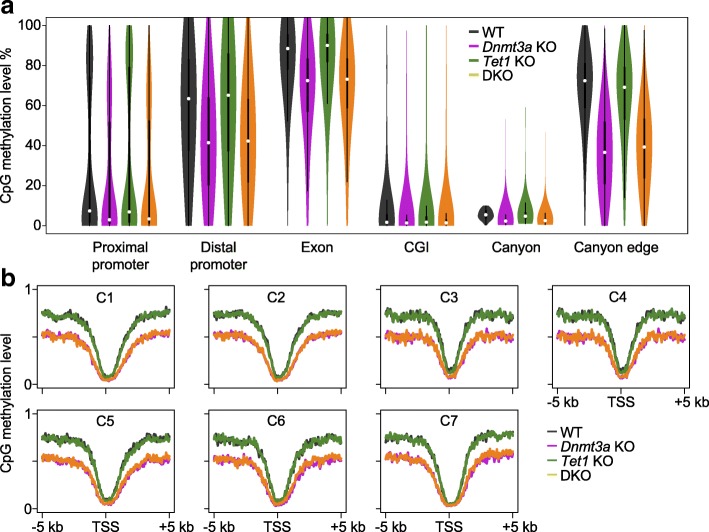
Analyses of DNA methylation in *Dnmt3a* KO, *Tet1* KO, and DKO ESCs. **a** *Violin plots* of CpG methylation levels in WT, *Dnmt3a* KO, *Tet1* KO, and DKO ESCs determined by WGBS at the indicated genomic features. **b** Average CpG methylation profiles at the flanking 5-kb regions of gene TSSs from clusters C1–C7 (listed in the same order as Fig. 6a) in WT, *Dnmt3a* KO, *Tet1* KO, and DKO ESCs

It has been shown that the H3K27me3 mark is lower genome-wide or more broadly distributed in a background with DNA hypomethylation [33, 34]. To examine this here, we performed DNA methylation analysis at single-base resolution in *Dnmt3a* KO, *Tet1* KO, DKO, and WT cells by WGBS. Similar levels of DNA methylation were observed between WT and *Tet1* KO cells and between *Dnmt3a* KO and DKO cells at different genomic features and DEG clusters (Fig. 7a, b). However, *Tet1* KO and DKO cells possessed lower H3K27me3 enrichment (at least at C1 and C2) compared to WT and *Dnmt3a* KO, respectively (Fig. 6b and Additional file 1: Figure S10b). These data suggest that methylation-independent mechanisms may also be involved in the synergistic regulation of H3K27me3 by DNMT3A and TET1.

Taken together, in this work we showed DNMT3A and TET1 regulate gene expression not only through controlling DNA methylation but also via regulating the histone landscapes at the promoters they bind. These data help resolve the conundrum of the commonly observed lack of correlation between changes in DNA methylation and changes in gene expression by demonstrating that the influence of DNA methylation is mediated proximally via histone mark changes and thus is dependent both on the initial methylation state and the broader epigenetic context.

## Discussion

The maintenance and dynamic regulation of DNA methylation in stem cells and during normal development remains the subject of much debate and research. Here we have demonstrated that DNA methyltransferase DNMT3A and dioxygenase TET1 synergistically regulate gene expression and epigenetic landscapes in mouse ESCs, via their complementary binding profiles and functional interplay.

This study first posits that DNMT3A, as opposed to DNMT3B, is the predominant de novo methyltransferase in establishing and maintaining DNA methylation patterns in mouse ESCs (Fig. 1), similar to its key role in HSCs [9]. Analysis of chromatin binding of DNMTs showed that DNMT3A1 and 3B1 have distinct binding targets, while DNMT3A2 shares binding regions with both 3A1 and 3B1. The different binding patterns of DNMT3A isoforms may result from the presence of 219 amino acids at the N-terminus of the longer DNMT3A1 isoform. While DNMT3A and 3B share high homology, and DNMT3B has several splicing isoforms, this N-terminal region is unique to DNMT3A1. The distinct binding pattern also suggests distinct functions in ESC maintenance and differentiation that will be worthy of future investigation. It will also be interesting to determine the presence and role of these isoforms in other tissues, such as the hematopoietic system and skin [35], in which DNMT3A has been shown to have a key functional role.

In addition, our data demonstrate that DNMT3A1 and TET1 have complementary binding patterns at key genomic regions (Fig. 3). Deletion of *Tet1* resulted in increased protein accessibility for DNMT3A1 but deletion of *Dnmt3a* had a modest but varied effect on TET1 binding, suggesting TET1 could serve as an anchor protein to establish boundaries for DNMT3A binding in order to limit DNA methylation (Fig. 5). Even though DNMT3A binding is extended after TET1 loss, it never fully occupies the core regions of promoters with high CpG density where TET1 originally binds. We suspect this is due to the continued presence of other factors, such as transcription factors and histone modifications, which contribute to excluding DNMT3A from occupying those high CpG-dense regions. In contrast, at the centers of TSSs that exhibit lower CpG density, where TET1 and H3K4me3 are absent, DNMT3A does bind. These findings suggest that local CpG density may be a key determinant of the complementary binding patterns between DNMT3A and TET1 (in addition to other factors) and therefore that the epigenetic landscape is encoded in the DNA sequence.

Regarding DNMT and TET interactions, we had previously reported that DNMT3A and TET2 compete and cooperate to regulate HSC functions [31]. Furthermore, during mouse early embryogenesis, the dynamic DNA methylation mediated by TETs and DNMT3s is crucial to regulation of the *Lefty-Nodal* signaling pathway in body plan formation [36]. Here we bring together these observations by demonstrating that not only DNMT3A (especially DNMT3A1) but also TET1, impacts histone modifications, ultimately influencing gene expression (Fig. 6). As discussed above, they may function together to poise the expression of bivalent genes largely via PRC2-mediated H3K27me3 modification, which is critical for the repression of developmental genes. This concept is also consistent with the reports that loss of DNMT3A and DNMT3B regulates H3K27me3 but has no impact on H3K4me3 [37] and that TET1 is required for chromatin binding of EZH2 in mouse ESCs [15]. This phenomenon may occur during ES cell differentiation: TET1 is gradually lost while DNMT3A is dynamically regulated, affecting H3K27me3 and leading to expression of developmental genes. Further investigation is needed to uncover the precise mechanism by which DNMT3A and TET1 influence PRC2 complex activity.

One possibility is a direct protein–protein interaction between DNMT3A/TET1 and PRC components as discussed above. Alternatively, DNMT3A/TET1 may facilitate polycomb enrichment indirectly, through affecting chromatin accessibility. For example, loss of DNMT3A1 could enable local chromatin changes which even modestly permit more transcription factors binding, adjacent to bivalent promoters. Then, such transcription factors would result in reduced polycomb. It will be important to ultimately understand these specific interactions in future studies.

Recently, some of these findings were corroborated using a similar in vivo biotinylation system in murine ES cells [26]. DNMT3A1 was shown to preferentially binds to the edges of H3K27me3-rich bivalent CpG islands, as we see also with canyons. These ESC data are consistent with a model in which DNMT3A1 is mediating turnover of DNA methylation at CpG island shores in the presence of TET1, indicating a dynamic relationship between the two antagonizing proteins.

While the work here is focused on ESCs, these findings may have relevance for the role of DNMT3A and TET2 in hematologic malignancies. Counterintuitively, they have been found co-mutated in some acute myeloid leukemias and T cell lymphoma subtypes [38]. This suggests an oncogenic cooperation between DNMT and TET deficiency that may involve deregulation of DNA methylation and histone modification, specifically at some key loci. Our work could provide additional valuable insights into many of the remaining questions surrounding the role of DNMT and TET proteins in maintaining epigenetic landscapes and gene regulation in a variety of cell types and malignancies.

## Conclusions

Using genetically engineered mouse ESCs as a model, here we reveal the synergistic regulation of epigenetic landscapes by DNMT3A and TET1. We found DNMT3A, especially the longer isoform DNMT3A1, exhibits enrichment at distal promoters and canyon edges, but is excluded from TSSs and canyons where TET1 binds. Knockout experiments suggested that TET1 may play a critical role in defining methylation boundaries by protecting the proximal promoters and canyons from DNMT3A. We demonstrate that DNMT3A and TET1 impact histone modifications which in turn regulate gene expression dynamically. With their binding interplay, they synergistically constrain gene expression from bivalent promoters by regulating PRC2-mediated H3K27me3 enrichment.

## Methods

### Cell lines and cell culture

Mouse ESCs were cultured on 0.1% gelatin-coated dishes in DMEM (Gibco, 11,965–092) supplemented with 15% fetal bovine serum (Foundation, 900–108), 1 × non-essential amino acids (Gibco, 11,140–050), 1 × GlutaMAX (Gibco, 35,050–061), 1 μM 2-Mercaptoethanol (Sigma, M7522), 1 × sodium pyruvate (Sigma, S8636), 1 × Penicillin/Streptomycin (Gibco, 15,140–122), and 1000 U/mL LIF (Millipore, ESG1106).

Bio-*Dnmt3a1* ESCs were obtained by cloning *Dnmt3a1* cDNA (BC007466; Open Biosystems) into pENTR/D-Topo vector (Thermo-Fisher) followed by Gateway recombination into p2Lox and inducible cassette exchange as previously described [23]. The generated cell line expresses an N-terminal Avi-tagged DNMT3A1 under control of a TetO promoter. Biotin tagging was performed in vivo by a ligase BirA encoded from the same vector. CRISPR/Cas9 deletion of *Tet1* in this cell line was achieved using pX330 and the guide RNA for exon 4 of *Tet1* was previously used [29].

To generate *Tet1*-FLAG cells, WT J1 ES cells were transfected with 1.0 μg eSpCas9-*Tet1* [39], 250 ng pRR-puro-*Tet1* [40], and 1.5 μg donor ssDNA (DNA sequence 5′-3′: CAATGTTGTTACCGTGTCCCCATACTCTCTCACTCATGTTGCGGGACCCTACAATAGATGGGTCGGCGGCAGTGGAGACTACAAAGACCATGACGGTGATTATAAAGATCATGACATCGATTACAAGGATGACGATGACAAGTAAGTTGGGTCTAAAGGCTTCTCTCATGTAATGCCTTTGCTAATGTGGTGTAGTGGGT) using Lipofectamine® 3000 reagent and selected by adding puromycin (2 μg/mL) to the ES medium 24 h post transfection. After 48 h of selection, surviving cells were trypsinized and plated at a colony density in normal medium on 10-cm dishes. After one week of culture, individual colonies were picked, expanded, and screened. To further delete *Dnmt3a* in established *Tet1*-FLAG ES cell line, exons 18–19 of *Dnmt3a* (*Dnmt3a1*) were deleted by dual sgRNAs-facilitated CRISPR/Cas9 system. Guide RNAs were cloned into pX459 vector and transfected into *Tet1*-FLAG J1 ES cells with Lipofectamine® 3000 reagent. The transfected cells were selected, cultured, and screened as above.

*Tet1* KO and *Dnmt3a*-*Tet1* DKO J1 ES cells were obtained by deleting exon 4 of *Tet1* alone or together with exons 18–19 of *Dnmt3a (Dnmt3a1)* using dual sgRNAs-facilitated CRISPR/Cas9. All guide RNAs and genotyping primers used in this paper are listed in Additional file 2: Table S1.

### Biotin-streptavidin immunodetection

After a 16 h doxycycline (2 μg/mL) induction, whole-cell extracts were obtained from 5 × 10^6^ Bio-*Dnmt3a1* ES cells by resuspension in CytoBuster Protein Extraction Reagent (Millipore) followed by 15-min incubation at room temperature (RT). Non-induced ESCs served as a negative control. Samples were spun at 20,000 g, 4 °C for 15 min and supernatant was isolated. Immunoprecipitation of biotinylated-DNMT3A1 was performed with M280-streptavidin beads (Thermo Fisher) using whole-cell extract from cultures with or without doxycycline induction. Equal amounts of protein were resuspended in Laemmli buffer and loaded for western blot analysis. The following antibodies were used: anti-DNMT3A (Santa Cruz, sc-20,703), anti-TET1 (provided by Dr. Guoliang Xu, [24]), and anti-β-actin (Santa Cruz, sc-47,778).

### Chromatin immunoprecipitation

Biotin-Streptavidin ChIP was performed as described, with some modifications [41]. After a 16-h doxycycline (2 μg/mL) induction, 5 million cells were fixed for 10 min with 1% formaldehyde, followed by chromatin extraction and sonication to generate 200–500 bp fragments. Ten percent of chromatin was kept as input. Immunoprecipitation was performed overnight at 4 °C using M280-streptavidin (Thermo Fisher) magnetic beads. ChIP washes were performed in the following order: 2% SDS, 1 × high salt buffer (20 mM Tris-HCl pH 8.0, 0.1% SDS, 2 mM EDTA, 500 mM NaCl, 1% Triton X-100), 1 × LiCl buffer (10 mM Tris-HCl pH 8.0, 1% NP40, 1 mM EDTA, 0.25 M LiCl, 1% NaDOC), 1 × TE with 0.2% Triton X-100, and 1 × TE buffer. Cross-linking was reversed with the addition of elution buffer (20 mM Tris-HCl, pH 7.5, 5 mM EDTA, 50 mM NaCl, 1% SDS, 50 μg/mL Proteinase K) to washed beads and incubation at 68 °C with rotation for 3 h. DNA was purified by MinElute PCR purification Kit (Qiagen).

FLAG (Sigma, F1804), TET1 [24], and SUZ12 (Abcam, ab12073) ChIP were performed in a similar procedure with 10 million cells. After incubation of chromatin and Dynabeads Protein A-antibody complex overnight at 4 °C, washes were done in the following order: 1 × low salt buffer (20 mM Tris-HCl pH 8.0, 0.1% SDS, 2 mM EDTA, 150 mM NaCl, 1% Triton X-100), 2 × high salt buffer (same as above), 2 × LiCl buffer (same as above), and 1 × TE buffer. Then cross-linking was reversed at 68 °C and immunoprecipitated DNA was purified by MinElute PCR purification Kit (Qiagen).

H3K4me3 (Millipore, 07–473) and H3K27me3 (Millipore, 07–449; Cell signaling, 9733S) ChIP were performed as previously described [6], with 3 million cells. For ChIP normalization, 50 ng spike-in chromatin (Active Motif, 53,083) and 2 μg spike-in antibody (Active Motif, 61,686) were added in each reaction before overnight incubation.

### ChIP-seq library preparation and high-throughput sequencing

For ChIP-seq, sequencing libraries were prepared using ThruPLEX DNA-seq 48D kit (Rubicon Genomics) following standard protocols. Samples with different dual-index barcodes were combined at equal molar ratios and sequenced as pools. Sequencing of library pools was performed on Illumina HiSeq 2000 or NextSeq 500 system according to Illumina standards, with 50-bp or 75-bp dual-end sequencing. Library de-multiplexing was performed following Illumina standards.

### Whole-genome bisulfite library preparation and sequencing

WGBS library construction was done as previously published [6]. For WGBS library construction, 2 μg of genomic DNA were isolated from ESCs and fragmented using Covaris S2 sonicator. After DNA fragmentation, libraries were constructed using the Illumina TruSeq DNA sample preparation kit. After ligation, libraries were treated with bisulfite using the EpiTect Bisulfite kit (Qiagen). Ligation efficiency was tested by PCR using TruSeq primers and PfuTurbo Cx Hotstart DNA polymerase (Stratagene). After determining the optimal number of PCR cycles for each sample, a large-scale PCR reaction (100 μL) was performed. PCR products were sequenced with Illumina HiSeq sequencing systems.

### Anti-cytosine-5-methylenesulfonate-immunoprecipitation, library preparation, and sequencing

Anti-cytosine-5-methylenesulfonate-immunoprecipitation (anti-CMS-IP) was performed as previously described [42] with slight modification. Genomic DNA was fragmented using Covaris Focused-ultrasonicator (average fragment size is ~ 300 bp) and ligated with methylated adaptors using NEBNext® Ultra™ DNA Library Prep Kit for Illumina® following the manufacturer’s instructions. The constructed libraries were treated with sodium bisulfite (QIAGEN). Purified bisulfite treated DNA libraries were then denatured for 10 min at 95 °C (0.4 M NaOH, 10 mM EDTA) followed by neutralized with the addition of equal volume of ice-cold 2 M ammonium acetate, pH 7.0. Denatured DNA libraries were incubated with antiserum to CMS in 1 × immunoprecipitation buffer (10 mM sodium phosphate, pH 7.0, 140 mM NaCl, 0.05% Triton X-100) overnight at 4 °C and precipitated with Protein A and protein G beads. Precipitated DNA was eluted with Proteinase K and purified by phenol-chloroform extraction. Purified DNA libraries were amplified by 10–12 cycles of PCR using KAPA HiFi HotStart Uracil polymerase. DNA sequencing was carried out using Illumina HiSeq sequencing systems.

### Global quantification of 5mC and 5hmC

Measurements of 5mC and 5hmC were performed by Zymo Research (http://zymoresearch.com) using high-pressure liquid chromatography coupled with mass spectrometry.

Dot blot analysis was performed as previously described [42]. Briefly, purified genomic DNA were denatured in 1 M NaOH, 25 mM EDTA at 95 °C for 10 min followed by neutralized with ice-cold 2 M ammonium acetate (pH 7.0). The denatured DNA were spotted on a nitrocellulose membrane in a Bio-Dot apparatus (Bio-Rad) with twofold serial dilution. Blotted DNA samples were washed with 2 × SSC buffer under filtered vacuum pressure system and then air-dried at RT for 10–15 min. The DNA were cross-linked with a membrane vacuum baker at 80 °C for 2 h. Next, the membrane was blocked with 5% non-fat milk at room temperature for 1 h and incubated with either anti-5-hmC primary antibody (1:5000, Active Motif, Cat# 39769) or anti-5mC antibody (1:1000, Millipore, Cat# MABE146) overnight at 4 °C. The membranes were washed with TBST buffer for 10 min three times then incubated with anti-rabbit secondary antibody (1:10,000, Sigma, Cat# 7074) for 1 h at RT. The membranes were then washed for 10 min three times and visualized by chemiluminescence with West-Q Pico Dura ECL Solution (Gendeport, Barker, TX, USA). To ensure the same loading amount of DNA, after visualization, the same membrane was washed with TBST and stained with 0.02% methylene blue in 0.3 M sodium acetate (pH 5.2) to ensure equal spotting of total DNA on the membrane.

### Whole genome bisulfite sequencing data analysis

We used BSMAP [43] to align the paired-end bisulfite treated reads to the mouse genome mm9 with the default parameters. BSeQC [44] was then used to remove the technical biases in WGBS data. First, we used the M-bias plot to determine the size of overhang, which would induce the artifact in end-repair process, then remove three bases from the repair end. Second, we removed the clonal reads from the PCR amplification based on a Poisson *P* value cutoff of 1 × 10^−5^. Third, we excluded one overlapping segment of two read mates to avoid over double counting. After removing the technical biases, we used MOABS [45] to calculate the methylation ratio, identify the hypomethylated regions for one sample and differential methylated regions between paired samples. The methylation ratios were measured as the number of unconverted CpGs divided by all the covered read numbers for each CpG in the mCall module. The differentially methylated cytosines (DMCs) were defined with the credible difference cutoff 0.2 for CpGs with at least three reads; more than three consecutive CpGs with the same hypo- or hypermethylation state within 300 bp were merged as DMRs in mComp module. The UMRs with methylation ratio < 10% were detected by using Hidden Markov Model in mOne module. UMRs < 1 kb in length were excluded in this manuscript. The UMRs in this study were defined as ≥ 1 kb but < 3.5 kb. UMRs ≥ 3.5 kb were defined as “canyons.”

### CMS-seq, histone modification, and TET1 ChIP-seq data analysis

The raw reads of the 5-hydroxymethylation CMS samples, paired-end 100 bp long, were mapped to the mouse genome mm9 using BSMAP [43]. All the raw reads of histone modification and TET1 ChIP-seq data were mapped to mouse genome mm9 using Burrows-Wheeler Alignment tool (BWA) [46]. Reads that could be mapped to multiple locations were removed. In order to remove the PCR resulted clonal reads, at most two clonal reads were kept for subsequent analysis. The number 2 was based on Poisson *P* value cutoff of 1 × 10^−5^ determined by the total number of reads with respect to the theoretical mean coverage across the genome. The remaining reads were then subject to the module Dtriple in DANPOS v2.2.1 [47] for read depth normalization, input signal subtraction, and the occupancy calculation. The TET1 peaks were called by MACS2 [48] with parameters “broad: True and broad-cutoff: 0.01.”

### Bio-DNMT3A/3B ChIP-seq data analysis

The Bio-DNMT3A1, DNMT3A2, and DNMT3B1 samples were sequenced at paired-end 100 bp long. Because several studies have suggested that some DNMT3A binding sites are in repetitive regions, in order to mapping the reads to repetitive regions, the reads were mapped to the mouse genome mm9 using bowtie [49] by allowing up to two mismatches and 100 alignments for a read. We then used CSEM [50] to allocate the multi-reads using Expectation-Maximization. Similar to other ChIP-seq analysis, we kept two clonal reads to remove the PCR bias. The occupancy of each sample was calculated by the module Dtriple in DANPOS v2.2.1 [47] with the read depth normalization and input signal subtraction.

### Dynamic analysis of DNMT3A1, TET1, and histone marks relative to *Dnmt3a* KO, *Tet1* KO or *Dnmt3a-Tet1* double KO (DKO)

We used DANPOS v2.2.1 [47] to quantitatively compare occupancy signal between ChIP-seq samples, with considering the sequencing depth and ChIP-seq input occupancy signal. For H3K4me3 and H3K27me3 spike-in ChIP-seq, we normalized the occupancy signal with factors calculated from *Drosophila* sequence tags in each sample. DANPOS v2.2.1 calculated the occupancy difference at ten bases between *Dnmt3a* KO/*Tet1* KO and WT. For each ten base pairs, the differential value was measured by the absolute log_10_ *P* value. The positive value means the occupancy is greater in *Dnmt3a* KO/*Tet1* KO relative to WT, and vice versa.

### DNMT3A/3B genome enrichment

In order to find genome-wide enriched binding sites of DNMT3A/3B, we partitioned the mouse genome to 1-kb windows. First, we removed the windows containing satellite repeats to avoid a false positive caused by the variant repeat number among mouse strains [21]. Furthermore, windows without covered by WGBS data and ChIP-seq input data were removed from subsequent analysis. All the remaining windows were used to identify the statistically enriched regions using the module Dregion in DANPOS v2.2.1 [47]. These enriched regions were used to calculate the distribution of genomic features.

### RNA-seq data analysis

Paired-end reads were sequenced for RNA-seq. We used Trim Galore (http://www.bioinformatics.babraham.ac.uk/projects/trim_galore/) to trim the low-quality bases and the adapters. TopHat [51] was used to mapping the raw reads on mouse genome mm9 with the default parameters. The gene annotation used for transcriptome alignment is RefSeq downloaded from the UCSC Table Browser (genome.ucsc.edu/cgi-bin/ hgTables?command = start). SAMtools [52] was used in processing the output files of TopHat. Differentially expressed genes were identified by pairwise comparison using the software Cufflinks [51] with cutoff: FDR ≤ 0.05. Average log-transformed gene scaled FPKM expression values of all the differentially expressed genes were used to perform the unsupervised clusters (K-means) and plot the heatmap. Based on the TSS order from the clustering result, we retrieved the normalized occupancy values and the differential values (relative to WT) of TET1, DNMT3A1, H3K27me3, and H3K4me3 across flanking the 5-kb region around gene TSSs, which were then subjected to TreeView to plot the heatmap figures.

### Clustering analysis of DNMT3A1, DNMT3A2, and DNMT3B1 occupancy

In order to measure the different binding properties of DNMT3A1, 3A2, and 3B1 around gene TSSs, we selected the longest isoform for each gene resulting in 22,766 unique gene TSS locations. We then retrieved the normalized occupancy values of DNMT3A1, 3A2, and 3B1 in WT across the flanking 5-kb region around gene TSSs and clustered all TSSs into five groups using the K-Means R package. Based on the same order from K-means result, we: (1) retrieved the normalized occupancy values of TET1 (WT and *Dnmt3a KO*), DNMT3A1 (WT and *Tet1 KO*), H3K4me3, and H3K27me3 across the flanking 5-kb region around gene TSSs, which were then subjected to TreeView to plot the heatmap figures; (2) calculated CpG density for each gene promoter (− 1 kb ~ + 0.5 kb); (3) calculated FPKM for each gene from RNA-seq analysis; and (4) plotted the average profiles of 5mC level and 5hmC level centered on TSSs.

## Additional files

Additional file 1: **Figure S1–S11.** Figures and legends for Supplementary Figures S1–S11. (PDF 15.6 Mb)

Additional file 2: **Table S1.** List of oligonucleotides: guide sgRNAs and primers. (PDF 52 Kb)
