# Supplementary material for: DNMT3A and TET1 cooperate to regulate promoter epigenetic landscapes in mouse embryonic stem cells
Source: Genome Biol. 2018 Jul 12;19:88. doi: 10.1186/s13059-018-1464-7 (PMC6042404; doi:10.1186/s13059-018-1464-7)
Supplement: Supplementary file 1 — Figure S1–S11. Figures and legends for Supplementary Figures S1–S11. (PDF 15.6 Mb) [file 13059_2018_1464_MOESM1_ESM.pdf]

Additional file 1: Figure S1-S11

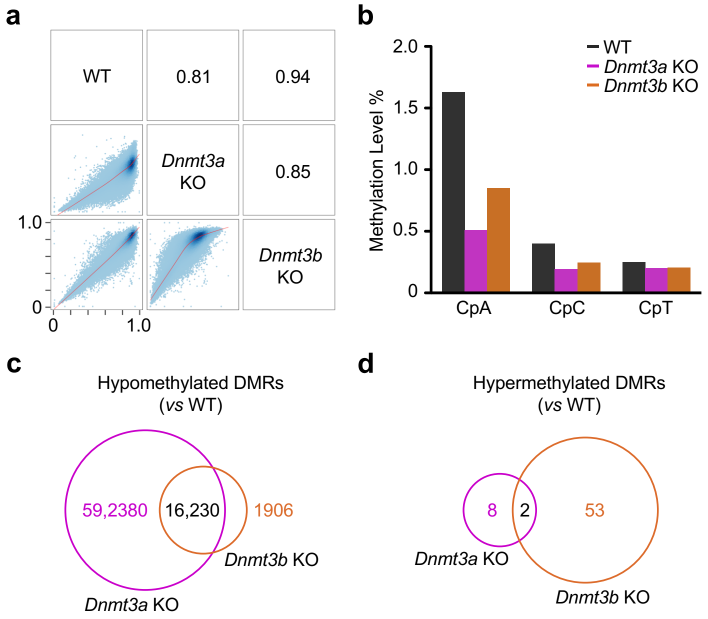

Figure S1. DNA methylation loss in *Dnmt3a* KO and *Dnmt3b* KO mouse ESCs

(**a**) Paired Pearson's correlation of the genome-wide methylation of WT, *Dnmt3a* KO and *Dnmt3b* KO ESCs. Each blue dot in the three lower-left plots represents a 1 kb window in the genome, and is plotted on the overall methylation level of each paired sample. The red lines in the lower triangular plots are the LOWESS smoothing curves calculated by R. The values in the upper-right boxes are the correlation coefficients between paired samples. (**b**) Average genome-wide methylated cytosine percentage at CpA, CpC and CpT dinucleotides compared between WT, *Dnmt3a* KO, *Dnmt3b* KO J1 ESCs determined by WGBS. (**c-d**) Venn diagrams illustrate the number of hypomethylated (**c**) or hypermethylated (**d**) differentially methylated regions (DMRs) in *Dnmt3a* KO and *Dnmt3b* KO ESCs, compared to WT ESCs, and those that overlap.

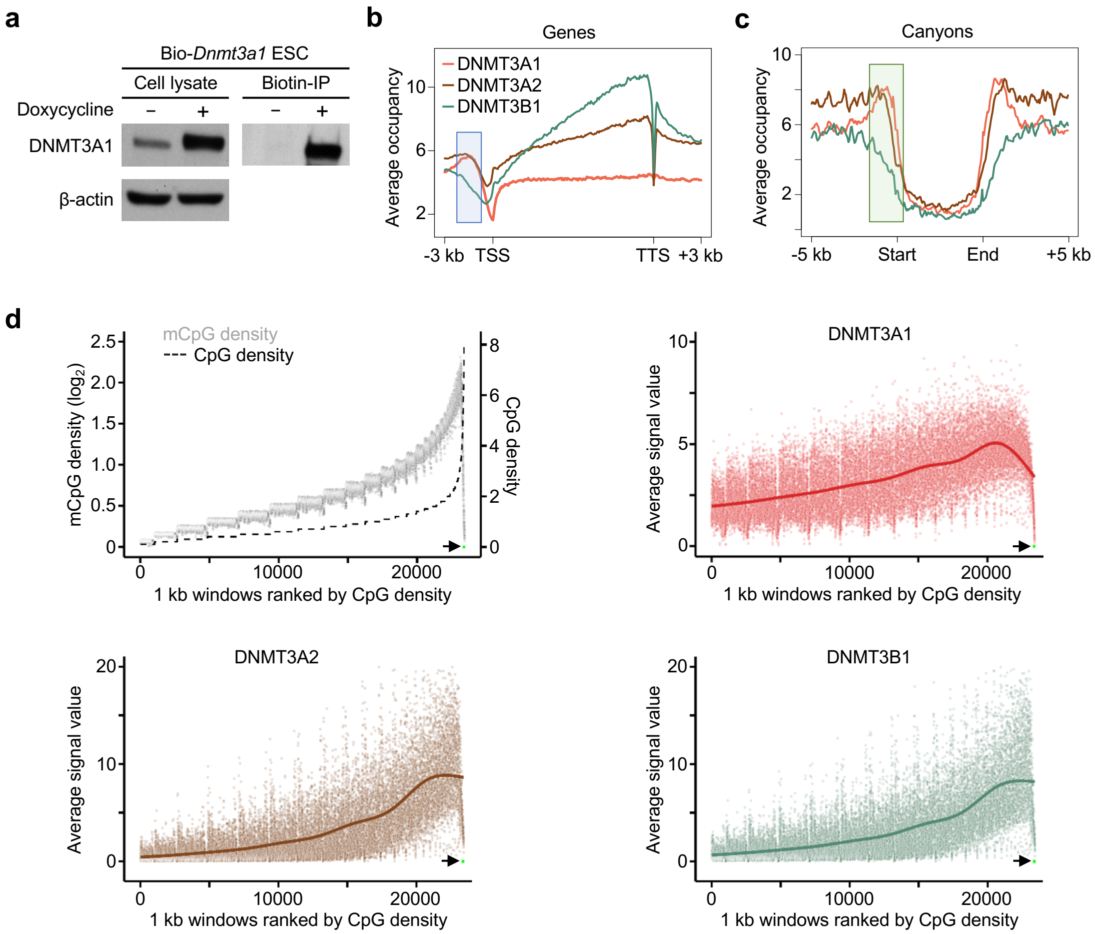

Figure S2. Distinct binding profiles of DNMT3A1, DNMT3A2 and DNMT3B1 in WT ESCs

(**a**) Western blots against DNMT3A showing expression of DNMT3A1 in Bio-*Dnmt3a1* ESCs after doxycycline induction and immunoprecipitation with streptavidin-coated beads. (**b-c**) Binding profiles of DNMT3A1, DNMT3A2 and DNMT3B1 across all gene bodies (**b**) and canyons (**c**), normalized to same length (Start-End). Highlighted region are distal promoter and canyon edge, where both DNMT3A1 and 3A2 binding is enriched. (**d**) CpG density, methylated CpG (mCpG) density and enrichments for DNMT3A1, DNMT3A2 and DNMT3B1 at 1 kb genomic windows ranked by CpG density. Windows overlapping with CpG islands are indicated as a green square (right arrow). Smoothing was applied using 100 consecutive windows.

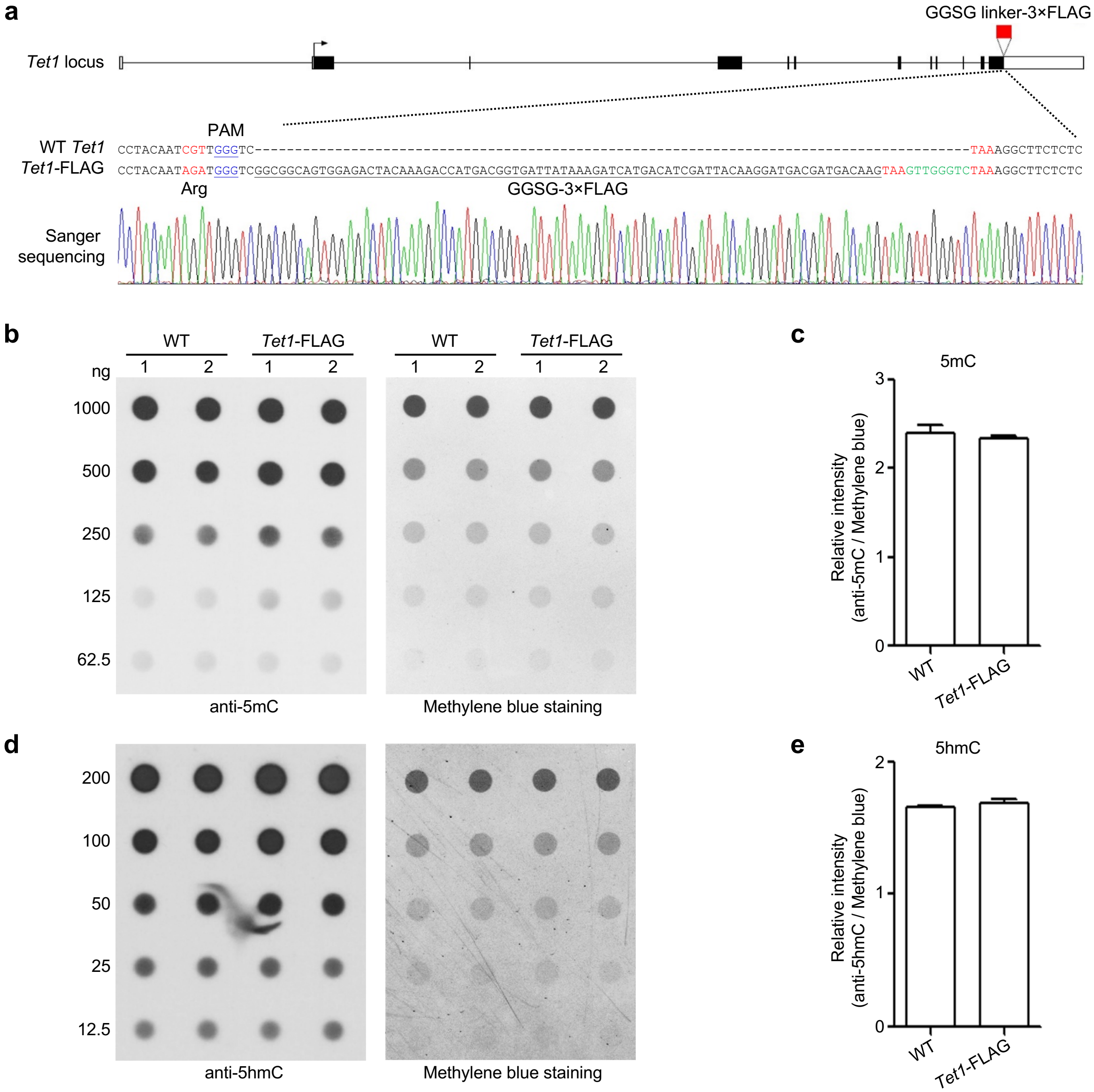

Figure S3. Generation and 5mC/5hmC quantification for *Tet1*-FLAG ESCs

(**a**) Generation of *Tet1*-3×FLAG (*Tet1*-FLAG) J1 ESCs by CRISPR-Cas9 facilitated HDR. A GGSG linker-3×FLAG tag was added at the C terminus of *Tet1* on both alleles. The PAM motif of guide RNA is shown in blue and the CGT in 'seed sequence' of the guide RNA was mutated to AGA to block Cas9 cleavage in donor ssDNA. (**b**) 5mC Dot Blot on 2-fold serial dilutions of genomic DNA isolated from WT and *Tet1*-FLAG ESCs. 2 replicates for each cell type are shown. Methylene blue staining of membranes used for dot blot analysis serve as a DNA loading control. (**c**) Quantification for 5mC dot blot analysis in (**b**). (**d**) 5hmC Dot Blot on 2-fold serial dilutions of genomic DNA isolated from WT and *Tet1*-FLAG ESCs. (**e**) Quantification for 5hmC dot blot analysis in (**d**).

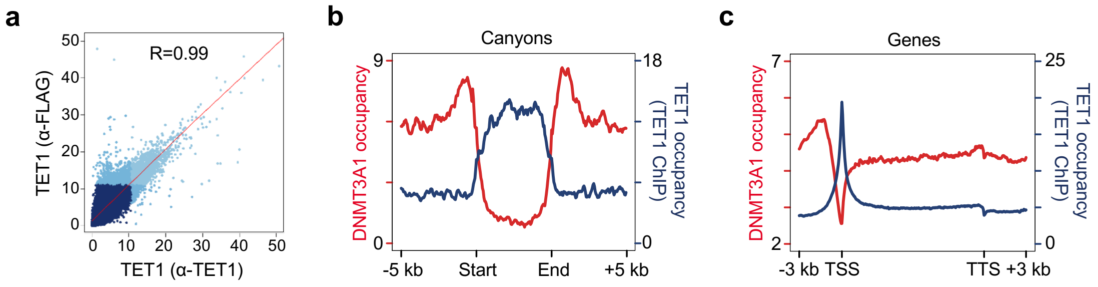

Figure S4. DNMT3A1 binding is complementary to TET1.

(**a**) Pearson correlation between anti-TET1 and anti-FLAG ChIP-seq data within *Tet1*-FLAG ESCs. Each blue dot represents binding occupancy in 10Kb windows across entire mouse genome. The red line is the LOWESS smoothing curves and the correlation coefficient between the two sets was shown. (**b**) Average binding occupancy of DNMT3A1 and TET1 (anti-TET1 ChIP-seq) across all canyons, normalized to same length (Start-End), and 5 kb flanking regions. (**c**) Average binding occupancy of DNMT3A1 and TET1 (anti-TET1 ChIP-seq) across all gene bodies and 3 kb flanking regions.

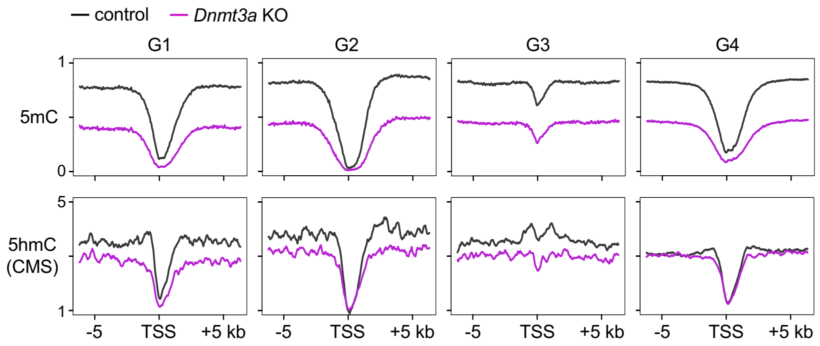

Figure S5. 5mC and 5hmC quantification in WT and *Dnmt3a* KO ESCs.

Average methylation level (5mC, up) and occupancy of 5hmC (anti-CMS, bottom) in genomic DNA of WT and *Dnmt3a* KO ESCs, at genes from each group defined in Fig. 4a.

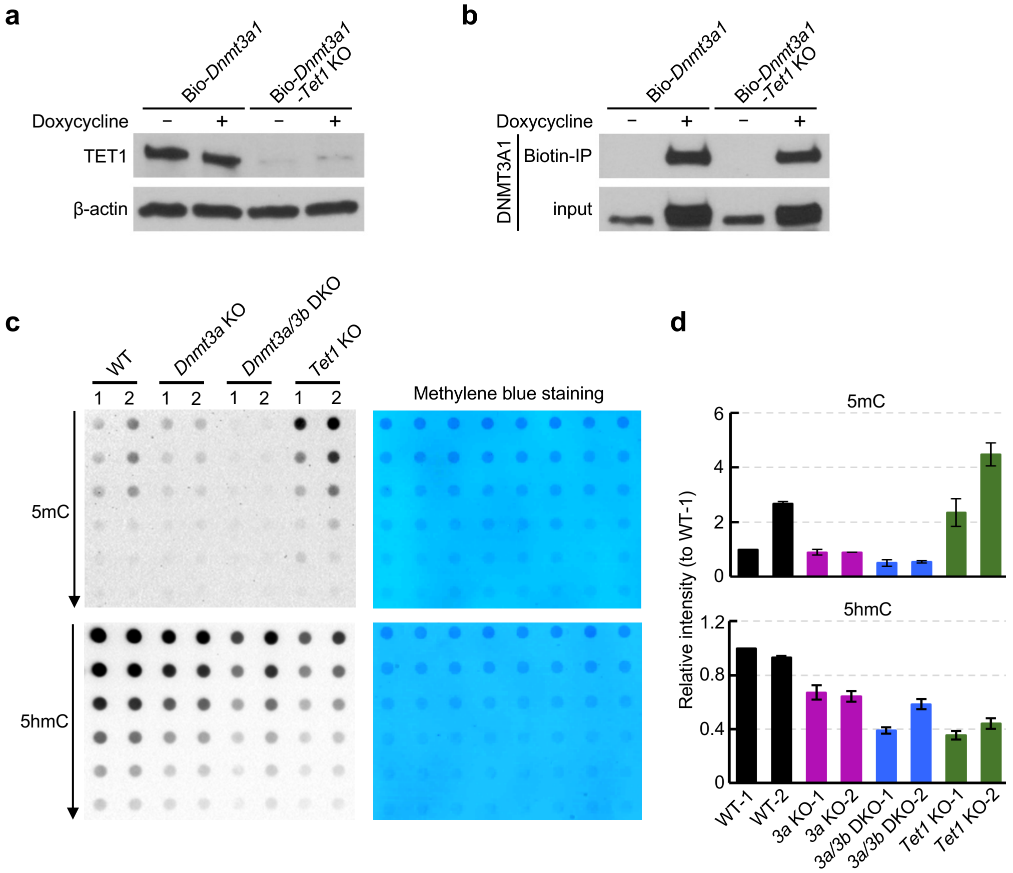

Figure S6. Generation and 5mC/5hmC measurement of *Dnmt3a* KO and *Tet1* KO ESCs

(**a**) Western Blot confirmation of *Tet1* KO in the Bio-*Dnmt3a1* ESCs. (**b**) Confirmation of expression of biotinylated DNMT3A1 after doxycycline induction in Bio-*Dnmt3a1* - *Tet1* KO and control ESCs by both western blot of whole cell lysate (input) and immunoprecipitation (Biotin-IP) with streptavidin-coated beads. (**c**) Dot Blot on 2-fold serial dilutions of genomic DNA isolated from WT, *Dnmt3a* KO, *Dnmt3a/3b* DKO, and *Tet1* KO ESCs, stained for 5mC (top) and 5hmC (anti-CMS, bottom) levels. 2 replicates for each cell type are shown. Methylene blue staining of membranes used for dot blot analysis serve as a DNA loading control. (**d**) Quantification for dot blot analysis in (**c**) plotted as the intensity of 5mC (top) and 5hmC (CMS, bottom) relative to the intensity of WT replicate 1 (WT-1).

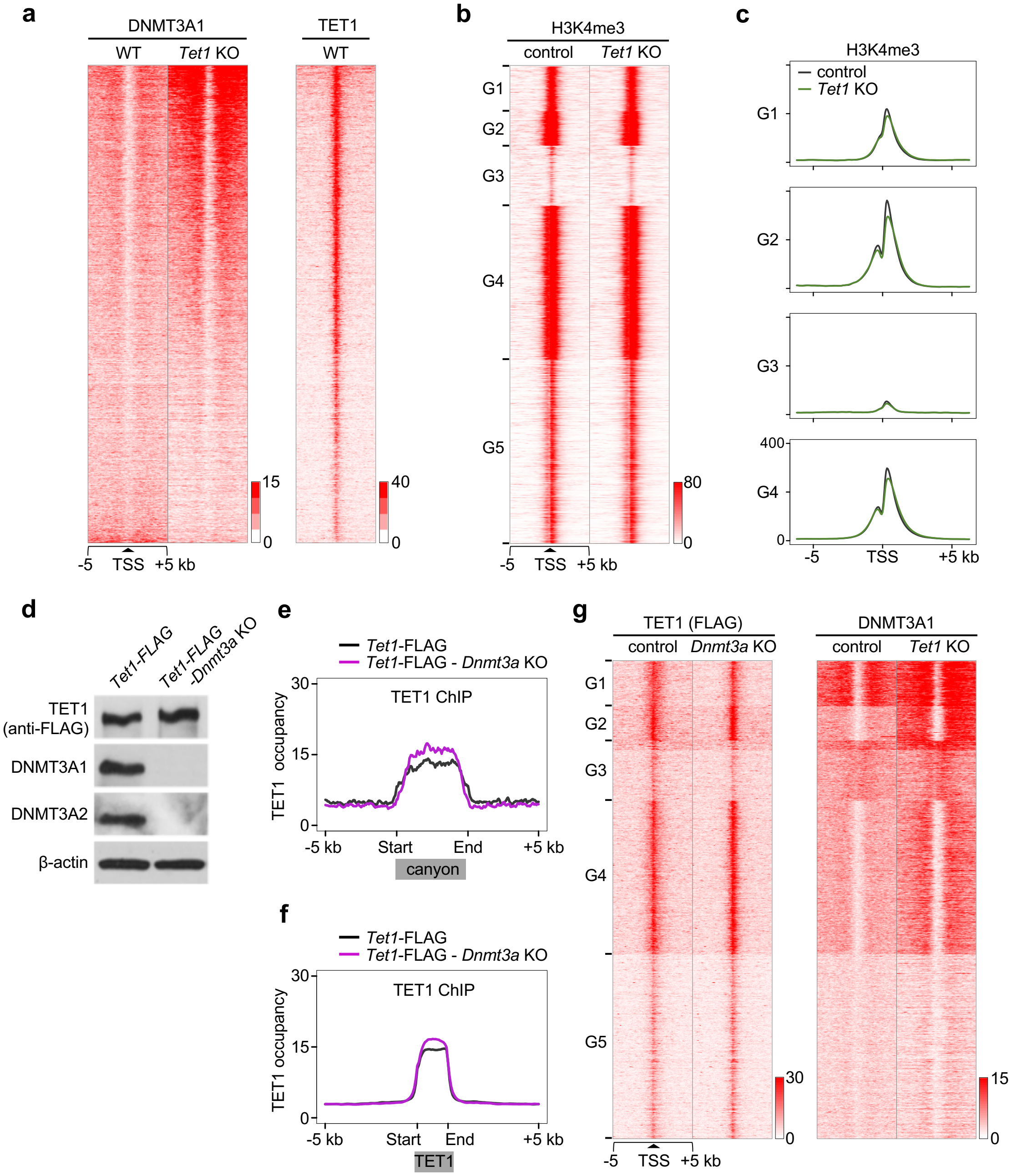

Figure S7. DNMT3A and TET1 impact each other's binding

(**a**) Heatmap representations from left to right: DNMT3A1 binding in WT or *Tet1* KO Bio-*Dnmt3a1* ESCs and TET1 binding in WT *Tet1*-FLAG ESCs on the flanking 5 kb regions of all TSSs, ranked by the change of DNMT3A1 binding in response to *Tet1* KO. (**b**) Heatmaps showing H3K4me3 enrichment in WT control and *Tet1* KO ESCs around all TSSs, listed in the same order as Fig. 4a. (**c**) Average density profiles for H3K4me3 around TSSs of genes from group G1-G4 in control and *Tet1* KO ESCs. (**d**) Western blots against TET1 (anti-FLAG), DNMT3A1 (Santa Cruz, H-295) and DNMT3A2 (Abcam, 64B1446), confirming the expression of TET1-FLAG and *Dnmt3a* KO in *Tet1*-FLAG ESCs. (**e**-**f**) The average occupancy of TET1 (anti-TET1 ChIP-seq) in *Tet1*-FLAG and *Tet1*-FLAG *Dnmt3a* KO ESCs across all canyons (**e**)

and TET1 peaks (**f**). (**g**) Heatmaps showing TET1 binding (left) in *Tet1*-FLAG and *Tet1*-FLAG - *Dnmt3a* KO ESCs and DNMT3A1 binding (right) in WT (Bio-*Dnmt3a1*) and *Tet1* KO ESCs around all TSSs, listed in the same order as Fig. 4a.

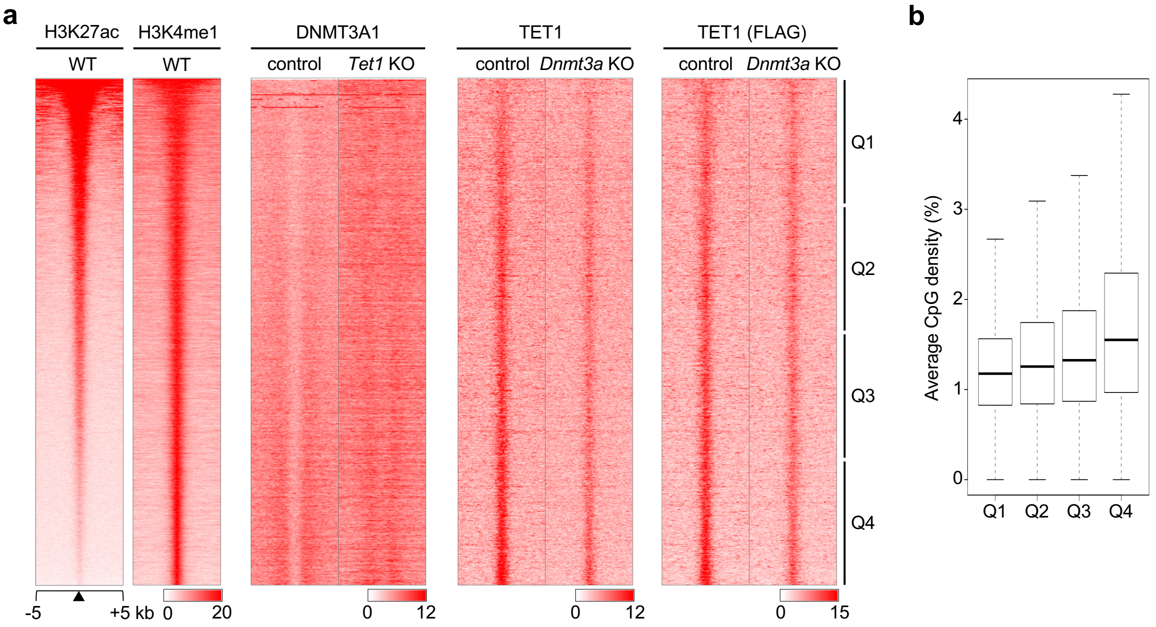

Figure S8. Decreased TET1 occupancy at enhancers in *Dnmt3a* KO

(**a**) Heatmaps from left to right: H3K27ac and H3K4me1 enrichment in WT cells (data from King, A. D. et al., *Cell Reports*, 2016), DNMT3A1 occupancy in control and *Tet1* KO cells, and TET1 occupancy (anti-TET1 or anti-FLAG ChIP-seq) in control and *Dnmt3a* KO cells, on the flanking 5 kb regions of the center of enhancers, ranked by the signal intensity of H3K27ac. Enhancers were identified as genomic regions with H3K27ac or H3K4me1 peaks outside of Refseq promoters. (**b**) Boxplot of average CpG density (normalized to 100 bp) at enhancer regions quartered into Q1-Q4 from top to bottom.

a

C1 genes (n=546)

ECM-receptor interaction	4.2E-6
Hippo signaling pathway	6.1E-5
TGF-beta signaling pathway	9.8E-5

C2 genes (n=563)

Proteoglycans in cancer	1.8E-6
PI3K-Akt signaling pathway	3.1E-5
Focal adhesion	1.1E-4
Transcriptional misregulation in cancer	3.5E-4

C3 genes (n=390)

Biosynthesis of antibiotics	1.2E-4
Focal adhesion	3.3E-4
PI3K-Akt signaling pathway	8.6E-4

C5 genes (n=438)

Ribosome	1.2E-5
Biosynthesis of antibiotics	8.2E-4
Glycolysis / Gluconeogenesis	1.2E-3

C6 genes (n=318)

Biosynthesis of antibiotics	4.9E-8
Carbon metabolism	1.5E-7
Biosynthesis of amino acids	1.2E-6
Glycolysis / Gluconeogenesis	2.3E-5

C7 genes (n=455)

RNA degradation	9.1E-4
Nucleotide excision repair	9.5E-3
Signaling pathways regulating pluripotency of stem cells	1.6E-2

b

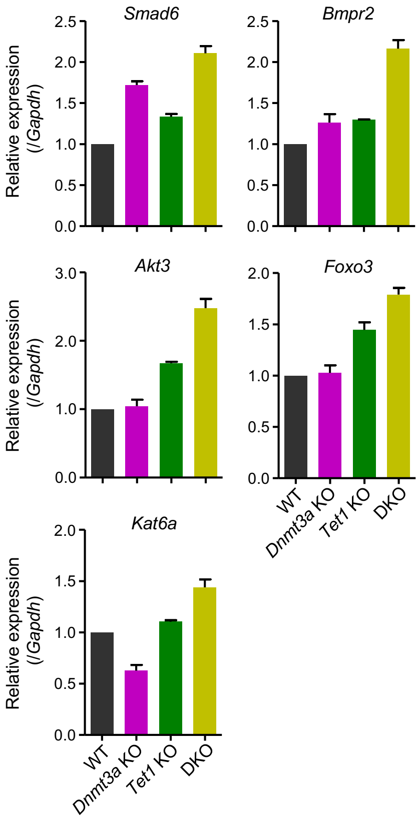

Figure S9. DNMT3A and TET1 regulate gene expression dynamically

(**a**) Functional annotation analysis (KEGG_ PATHWAY) of the genes from clusters 1-4 and 6-7. Each of the clusters analyzed here contain more than 300 genes. *P* values are listed on the right. (**b**) RT-qPCR confirmation of the expression change of *Smad6*, *Bmpr2*, *Akt3*, *Foxo3* and *Kat6a* genes determined by RNA-seq in *Dnmt3a* KO, *Tet1* KO and DKO ESCs. The expression level in WT ESCs was set as 1. Error bars represent standard error of the mean (SEM) from three independent experiments.

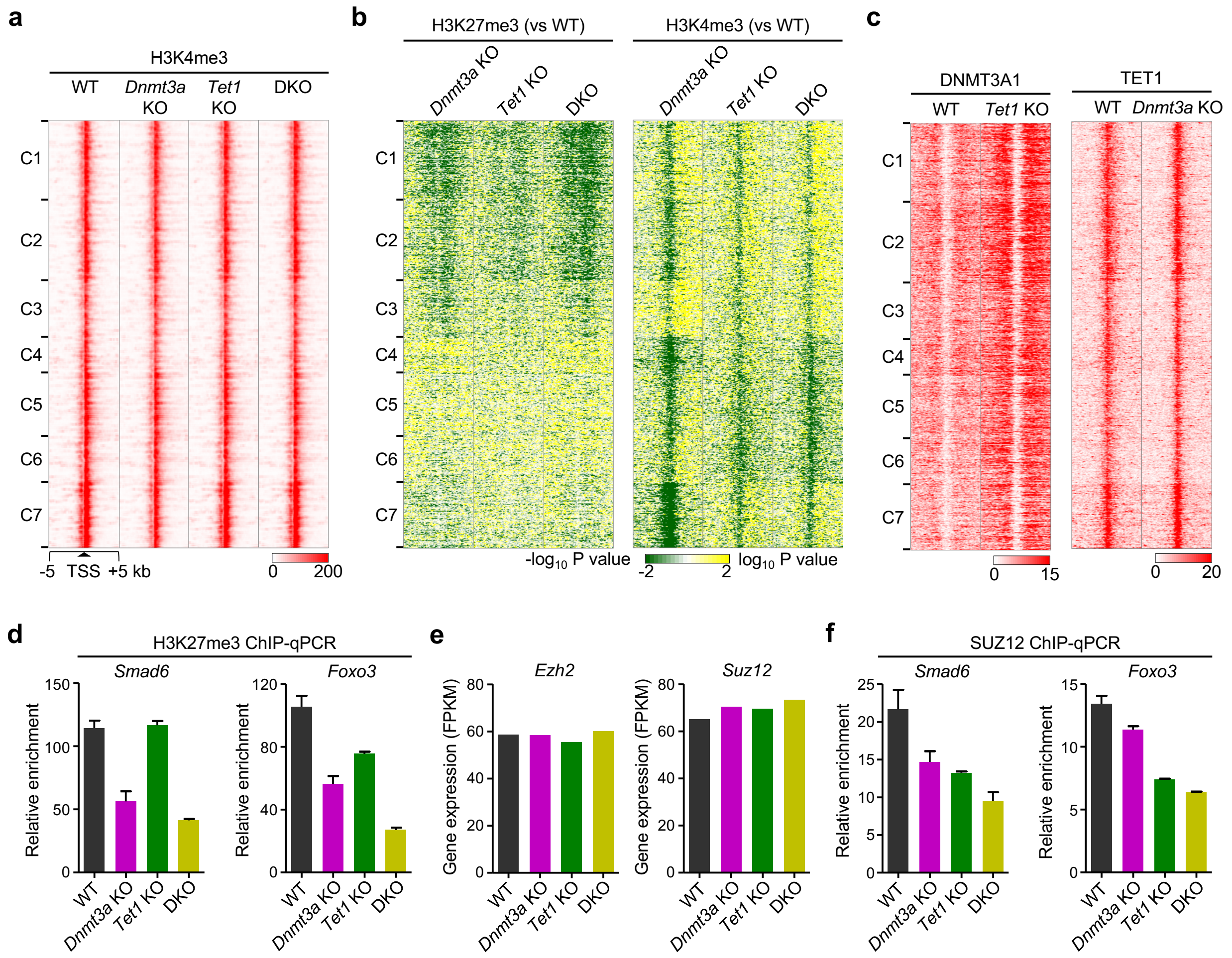

Figure S10. DNMT3A and TET1 regulate bivalent gene expression via PRC2-mediated H3K27me3 enrichment

(**a**) Heatmap representations of H3K4me3 enrichment on the flanking 5 kb surrounding the specific TSSs, listed in the same order as Fig. 6a, in WT, *Dnmt3a* KO, *Tet1* KO and DKO ESCs. (**b**) Heatmaps showing the signal difference of H3K27me3 and H3K4me3 between KO and control cells surrounding TSSs analyzed in clusters 1-7. Green, decreased; yellow, increased signal intensity, compared to WT control. (**c**) Heatmap representations of DNMT3A1 binding in *Tet1* KO and control ESCs and TET1 binding in *Dnmt3a* KO and control ESCs. (**d**) ChIP-qPCR showing reduced H3K27me3 over *Smad6* and *Foxo3* peak regions in *Dnmt3a* KO, *Tet1* KO and *DKO* ESCs, compared to WT control. (**e**) Expression levels (FPKM) of *Ezh2* and *Suz12* genes determined by RNA-seq in WT, *Dnmt3a* KO, *Tet1* KO and DKO ESCs. (**f**) ChIP-qPCR showing reduced SUZ12 enrichment over *Smad6* and *Foxo3* peak regions in *Dnmt3a* KO, *Tet1* KO and *DKO* ESCs, compared to WT control. H3K27me3 and SUZ12 ChIP-qPCR was normalized to a region at *Jmjd4* promoter, which lacks H3K27me3 in ESCs. Error bars represent standard error of the mean (SEM) from three independent experiments.

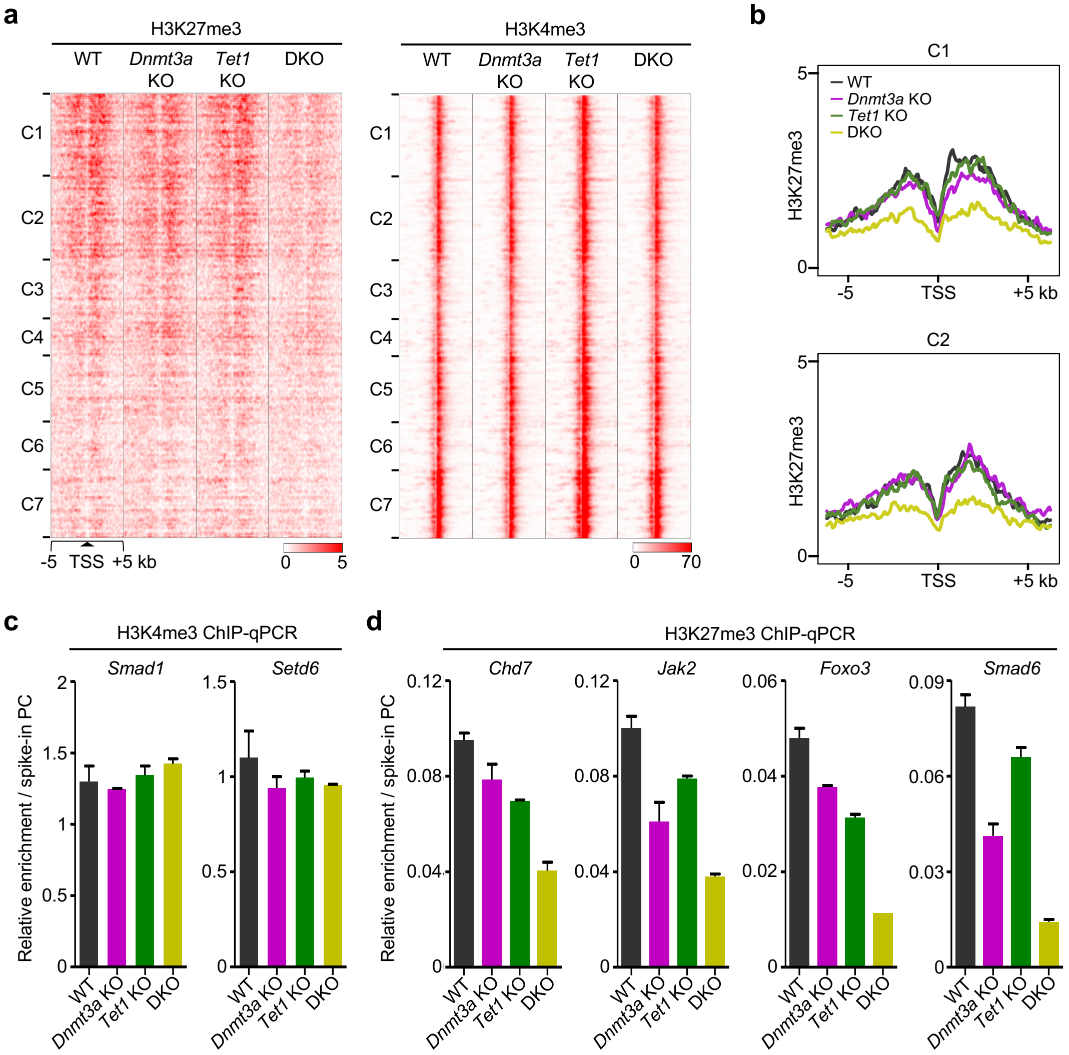

Figure S11. DNMT3A and TET1 regulating H3K27me3 enrichment at bivalent promoters was verified by spike-in histone ChIP-seq

(**a**) Heatmap representations of H3K27me3 and H3K4me3 enrichment (normalized to spike-in control) on the flanking 5 kb surrounding the specific TSSs in each cluster in WT, *Dnmt3a* KO, *Tet1* KO and DKO ESCs. (**b**) Average density profiles for H3K27me3 marks in (**a**) at genes from C1 (up) and C2 (bottom) clusters. (**c**) ChIP-qPCR showing similar level of H3K4me3 enrichment over *Setd6* and *Smad1* peak regions in indicated lines, normalized to a spike-in positive control (Active Motif, 53083). (**d**) ChIP-qPCR showing reduced H3K27me3 enrichment over *Chd7*, *Jak2*, *Smad6* and *Foxo3* peak regions. Error bars represent standard error of the mean (SEM) from two independent experiments.
