# Supplementary material for: DNMT3A and TET1 cooperate to regulate promoter epigenetic landscapes in mouse embryonic stem cells
Source: Genome Biol. 2018 Jul 12;19:88. doi: 10.1186/s13059-018-1464-7 (PMC6042404; doi:10.1186/s13059-018-1464-7)
Supplement: Supplementary file 2 — Table S1. List of oligonucleotides: guide sgRNAs and primers. (PDF 52 Kb) [file 13059_2018_1464_MOESM2_ESM.pdf]

# Additional file 2: Table S1

## Table S1. List of Oligonucleotides: guide RNAs and primers.

| Name | Sequence (5'-3') | Note |
|---|---|---|
| Tet1-Flag sg | TGCGGGACCCTACAATCGTT | guide RNA for *Tet1*-FLAG tagging |
| Tet1-Flag gt-F | TGATGTATCCCCCGAAGCCA | *Tet1*-FLAG ESCs genotyping primers |
| Tet1-Flag gt-R | CGGAGTTGAAATGGGCGAAAC | |
| Tet1KO sg-L | AGATTTCGTGGGGGTTACGG | guide RNAs for *Tet1* (exon 4) deletion |
| Tet1KO sg-R | TGAGGTCGGCTACGCCTATC | |
| Tet1KO gt-F | CCCTTCTCCCTCATTCAGCTC | *Tet1* KO genotyping primers |
| Tet1KO gt-R1 | TGTGTGGGTGTTTTCCCGAC | |
| Tet1KO gt-R2 | ATCCGTGGTGACTCTGGGTA | |
| 3aKO sg-L | TGTGGTAATGAGTTCTCGAT | guide RNAs for *Dnmt3a* (exon 18-19) deletion |
| 3aKO sg-R | TGTCTGCATTGCGGAGCTGG | |
| 3aKO gt-F | TCTGTGGCATCTCAGGGTGA | *Dnmt3a* KO genotyping primers |
| 3aKO gt-R1 | CCTCCAATCACCAGGTCGAA | |
| 3aKO gt-R2 | AGAGAGTGAGCACAGGCCAT | |
| Gapdh RT-F | AGGTCGGTGTGAACGGATTTG | RT-qPCR primers for *Gapdh* |
| Gapdh RT-R | TGTAGACCATGTAGTTGAGGTCA | |
| Smad6 RT-F | TTACACTGAAACCGAGGCCA | RT-qPCR primers for *Smad6* |
| Smad6 RT-R | TGGTCGTACACCGCATAGAG | |
| Bmpr2 RT-F | AAACCCGCAATCTCCCACC | RT-qPCR primers for *Bmpr2* |
| Bmpr2 RT-R | AGCGAATTGTGCCAACCTCA | |
| Akt3 RT-F | CGCACACGTTTCTATGGTGC | RT-qPCR primers for *Akt3* |
| Akt3 RT-R | GCTGCATCTGTGATCCCTTCT | |
| Foxo3 RT-F | GGTACCAGGCTGAAGGATCAC | RT-qPCR primers for *Foxo3* |
| Foxo3 RT-R | AGTCTCTGCTGGGTTAGGGAA | |
| Kat6a RT-F | TGTCTTTAGGGGCTTCAGCG | RT-qPCR primers for *Kat6a* |
| Kat6a RT-R | TTGCGGACTCTTCGTCTTCG | |
| K27me3-neg-F | GTTTCCCGGTGGATGGATCG | qPCR primers for H3K27me3/PRC2 ChIP negative control (*Jmjd4* promoter) |
| K27me3-neg-R | GTCAGCATAGGAGAAGGCGT | |
| Smad6 ChIP-F | CATGGATCACGATGGGCCG | qPCR primers for H3K27me3/PRC2 ChIP peak at *Smad6* promoter |
| Smad6 ChIP-R | CAGCCGCGCAACTTTTGAAG | |
| Foxo3 ChIP-F | CAGCAGCATGGCCGAATC | qPCR primers for H3K27me3/PRC2 ChIP peak at *Foxo3* promoter |

| | | |
|---|---|---|
| Foxo3 ChIP-R | GACGACGATGAAGACGACGA | |
| Chd7 ChIP-F | GGACAGTTCTTTCAAACCGGG | qPCR primers for H3K27me3 ChIP peak at *Chd7* promoter |
| Chd7 ChIP-R | GGGAAAGCCCTGTCTCTTACA | |
| Jak2 ChIP-F | TCTGGCCATGCTAAACGTCC | qPCR primers for H3K27me3 ChIP peak at *Jak2* promoter |
| Jak2 ChIP-R | GGGAGAGTTCTGAGGAATCGG | |
| Setd6 ChIP-F | CCCACACCTGTTCCATCAGC | qPCR primers for H3K4me3 ChIP peak at *Setd6* promoter |
| Setd6 ChIP-R | ACACCAGGTGAACGTCCAAG | |
| Smad1 ChIP-F | CTGGACACGCCCGACAAA | qPCR primers for H3K4me3 ChIP peak at *Smad1* promoter |
| Smad1 ChIP-R | GACCCGCGCTGAAGGAAATC | |
